# Potential Moderators of the Effects of Blood Flow Restriction Training on Muscle Strength and Hypertrophy: A Meta-analysis Based on a Comparison with High-Load Resistance Training

**DOI:** 10.1186/s40798-024-00719-3

**Published:** 2024-05-22

**Authors:** Yu Geng, Xueping Wu, Yong Zhang, Meng Zhang

**Affiliations:** 1 Department of Physical Education, Jiyang College of Zhejiang A&F University, Zhuji, 311800 People’s Republic of China; 2 School of Physical Education, Shanghai University of Sport, Shanghai, People’s Republic of China; 3 Department of Rehabilitation Medicine, School of Medicine, Shaoxing University, Zhejiang, People’s Republic of China; 4 School of Physical Education, Huzhou University, Zhejiang, People’s Republic of China

**Keywords:** Blood flow restriction training, High-load resistance training, Training effect, Strength, Hypertrophy, Training status, Protocol characteristics, Test specificity, Meta-analysis

## Abstract

**Background:**

While it has been examined whether there are similar magnitudes of muscle strength and hypertrophy adaptations between low-load resistance training combined with blood-flow restriction training (BFR-RT) and high-load resistance training (HL-RT), some important potential moderators (e.g., age, sex, upper and lower limbs, frequency and duration etc.) have yet to be analyzed further. Furthermore, training status, specificity of muscle strength tests (dynamic versus isometric or isokinetic) and specificity of muscle mass assessments (locations of muscle hypertrophy assessments) seem to exhibit different effects on the results of the analysis. The role of these influencing factors, therefore, remains to be elucidated.

**Objectives:**

The aim of this meta-analysis was to compare the effects of BFR- versus HL-RT on muscle adaptations, when considering the influence of population characteristics (training status, sex and age), protocol characteristics (upper or lower limbs, duration and frequency) and test specificity.

**Methods:**

Studies were identified through database searches based on the following inclusion criteria: (1) pre- and post-training assessment of muscular strength; (2) pre- and post-training assessment of muscular hypertrophy; (3) comparison of BFR-RT vs. HL-RT; (4) score ≥ 4 on PEDro scale; (5) means and standard deviations (or standard errors) are reported or allow estimation from graphs. In cases where the fifth criterion was not met, the data were requested directly from the authors.

**Results:**

The main finding of the present study was that training status was an important influencing factor in the effects of BFR-RT. The trained individuals may gain greater muscle strength and hypertrophy with BFR-RT as compared to HL-RT. However, the results showed that the untrained individuals experienced similar muscle mass gains and superior muscle strength gains in with HL-RT compared to BFR-RT.

**Conclusion:**

Compared to HL-RT, training status is an important factor influencing the effects of the BFR-RT, in which trained can obtain greater muscle strength and hypertrophy gains in BFR-RT, while untrained individuals can obtain greater strength gains and similar hypertrophy in HL-RT.

**Supplementary Information:**

The online version contains supplementary material available at 10.1186/s40798-024-00719-3.

## Key Points

Training status is an important factor influencing the effects of the BFR-RT.

Trained individuals may obtain greater muscle strength and hypertrophy gains in BFR-RT compared to HL-RT.

Untrained individuals may experience a smaller increase in strength and a similar increase in hypertrophy with BFR-RT compared to HL-RT.

**Supplementary Information:**

The online version contains supplementary material available at 10.1186/s40798-024-00719-3.

## Background

High-load resistance training (HL-RT) has long been considered as the “gold standard” protocol to increase muscle strength and mass. It has been suggested that ≥ 65% one-repetition maximum (1RM) is required to increase strength and hypertrophy [1–3]. However, mounting evidence indicates that the use of low-load resistance training (< 50% 1RM) combined with blood flow restriction (BFR-RT) results in strength and morphological responses [4]. The results of a previous study showed that low load resistance training with and without blood flow resulted in similar adaptations when sets of exercise were taken to failure [5], however the results of multiple studies showed the superiority of BFR-RT in terms of gains in muscle strength and hypertrophy when compared with similar low-load resistance training without blood flow restriction [6–8]. However, the literature is controversial about the magnitude of the adaptations when comparing BFR-RT to HL-RT. For example, some studies have reported greater increases in muscle strength for HL-RT when comparing to BFR-RT [9–13], while others have suggested similar gains between the two exercise protocols [14–17]. Moreover, some studies have reported that BFR-RT has higher muscle strength gains than HL-RT [18, 19]. Some studies compared the effects of BFR-RT and HL-RT through meta-analysis [20, 21]. In the meta-analysis by Lixandrão et al., they observed that BFR-RT and HL-RT have similar gains in muscle hypertrophy, while HL-RT is more effective in increasing muscle strength [20]. However, limited by the number of studies comparing BFR and HL-RT at that time, some important potential moderators (e.g., training frequency, etc.) could not be further explored [20].

The increase in muscle strength is the result of the coordination of nerve and muscle systems [22]. It is believed that neural adaptation dominates early in the training programme; later, as neural adaptations reach a plateau, muscular adaptation (hypertrophy) dominates [23]. At this stage, in intermediate and advanced training, progress is limited to the extent of muscular adaptation that can be achieved [23]. It has been believed that BFR-RT provides a potential time-effective approach to stimulate muscle adaptations [8, 24, 25], and even well-trained athletes may benefit from BFR-RT [26]. Thus, compared to HL-RT, training status and training duration may be a key factor affecting the effectiveness of BFR-RT. Additionally, the results of several studies have showed that compared with HL-RT, BFR-RT induced less hypertrophy in the proximal region [17, 27, 28]. Therefore, this regional specificity may be another influencing factor. Finally, HL-RT implies high-load exercise, which is similar to specific strength assessments (i.e., 1RM test), while during BFR-RT, participants are never exposed to high loads [29]. Thus, non-specific strength assessments (i.e., isometric or isokinetic tests) may more accurately reflect the response to the low-load training protocols [29]. In this regard, test specificity may also affect the results.

In short, the inconsistencies in the literature regarding effects of BFR-RT compared with HL-RT on the muscle strength and hypertrophy justify the need for synthesis and a comprehensive review of the available evidence. Therefore, based on previous studies, the purpose of this study was to conduct a meta-analysis comparing the responses of BFR-RT and HL-RT on muscle strength and hypertrophy. To further explore the effects on muscle strength and hypertrophy of these protocols, we will also consider potential influence factors such as population characteristics (i.e., training status, sex and age), protocol characteristics (i.e., upper or lower limbs, duration and frequency), test specificity (i.e., 1RM and spacing or equal speed testing), and region-specific adaptations in muscle mass.

## Methods

### Search Strategy and Study Selection

The articles were identified through the English databases Web of Knowledge, PubMed, EBSCO-SPORTDiscus from the earliest record up to February 2024, and the Chinese database WANFANG DATA, CNKI from the earliest record up to February 2024. The search strategy combined the English and Chinese terms (see Additional file 1: Table S1). Two reviewers (GY and ZY) evaluated the titles and abstracts of the retrieved articles to assessed their eligibility for the meta-analysis. In cases of differences, a consensus was adopted. If necessary, the third reviewer (WXP) evaluated the article. If the abstract did not provide sufficient information about the inclusion criteria, the reviewers read the full text.

### Eligibility Criteria

Studies were considered for inclusion if they met the following criteria: (1) articles were published in English or Chinese; (2) subjects were healthy people, (3) pre- and post-intervention assessment of muscular strength (i.e., dynamic, isometric or isokinetic test); (4) pre- and post-intervention assessment of muscle hypertrophy (i.e., magnetic resonance imaging, computerized tomography, or ultrasonography); (5) comparisons between HL-RT (i.e., > 65%1RM) and BFR-RT (i.e., < 50%1RM); (6) score ≥ 4 on the Physiotherapy Evidence Database (PEDro) scale.

### Study Quality

The quality of the study was determined by using the PEDro scale, based on the Delphi list [30]. Studies with a score ≥ 4 were included in this meta-analysis (see Additional file 1: Table S2). For each of the items (2–11) of the PEDro scale, two reviewers (GY and ZY) assessed the studies independently. In cases of disagreement, a consensus was adopted or a third reviewer (WXP) evaluated the study.

### Data Extraction

After screening of the studies, all included studies were assessed for eligibility based on their full texts. Two reviewers (GY and ZY) extracted data from the articles independently; in cases of disagreement, if no consensus could be reached, a third reviewer (WXP) was consulted. The data extracted were recorded relating to (1) population characteristics (i.e., age, sex and training status); (2) intervention protocol characteristics (i.e., duration, frequency, training load, volume, exercises etc.); (3) pre- and post-intervention assessment of muscle strength (i.e., dynamic, isometric, or isokinetic test); (4) pre- and post-intervention assessment of muscle hypertrophy (cross-sectional area [CSA], muscle thickness and muscle mass). The trained individuals were defined as athletes or individuals who participated in regular resistance training protocols before the intervention. The untrained individuals were defined as individuals who were sedentary or did not participate in regular resistance training protocols before the interventions. In cases of incomplete data availability, we extrapolated the data from figures or contacted the corresponding author. The graphical data were extracted using the OriginPro 2021 (Version 2021. OriginLab Corporation, Northampton, MA, USA) graphical digitizing tool. Only the last was included as the post-intervention value for analysis, when intervention effects were assessed at multiple time points. When intervention effects were measured through multiple measurement methods (e.g., CSA and muscle thickness for muscle size), the multiple outcomes were combined (i.e., using the mean of the outcomes) [31]. The combination was performed by Comprehensive Meta-Analysis software (version 3.3, Biostat, Inc., Englewood, NJ, USA). The extracted data of included studies are provided in Tables 1 and 2.

**Table 1:** Characteristics of studies included in the meta-analysis of muscle strength adaptations

References	Age	Training Status	N	Exercise	Protocol	Weeks (times/wk)	Exercise load	Occlusion pressure	Muscle strength assessment
Bjornsen et al. [26]	24	Trained	9	SQ	BFR-RT:30–15-12–8	2(5)	24–30%1RM	120mmHg	Dynamic SQ
									Isometric KE
	26		8		HL-RT:6–7 × 1–6	2(5)	74–76%1RM		
Buckner et al. [32]	18–35	Untrained	20	BC	BFR-RT1:4 × failure	8(2)	15%1RM	57mmHg	Dynamic BC
									Isometric BC
									Isokinetic BC (60°/s, 180°/s)
	18–35		20		BFR-RT2:4 × failure	8(2)	15%1RM	110mmHg	
	18–35		20		HL-RT:4 × failure	8(2)	70%1RM		
Centner et al. [33]	27	Untrained	M11	CR	BFR-RT:30–15-15–15	14(3)	20–35%1RM	50%AOP	Isometric CR
	26		M14		HL-RT:3 × 6–12	14(3)	70–80%1RM		
Centner et al. [28]	28	Untrained	M14	LP, KE, CR	BFR-RT:30–15-15–15	14(3)	20–35%1RM	50%AOP	Dynamic LP & KE
	28		M15		HL-RT:3 × 6–12	14(3)	70–85%1RM		
Clark et al. [14]	24	Untrained	9	KE	BFR-RT:3 × 30–50	4(3)	30%1RM	170mmHg	Isometric KE
	24		7		HL-RT:3 × 8–12	4(3)	80%1RM		
Cook et al. [34]	77	Untrained	12	KE, KF, LP	BFR-RT: ~ 3 × 30	12(2)	30%1RM	184 ± 25mmHg	Dynamic KE, KF & LP
									Isometric KE
	77		12		HL-RT: ~ 3 × 15	12(2)	70%1RM		
Cook et al. [35]	18–22	Untrained	6	KE, LP	BFR-RT:2 × 25,1 × failure	6(3)	20%1RM	180-200mmHg	Dynamic KE
									Isometric KE
	18–22		6		HL-RT:2 × 10,1 × failure	6(3)	70%1RM		
Davids et al. [36]	24	Trained	11	SQ, LP, KE, SSQ	BFR-RT:30 + 2–3 × 15	9(3)	30–40%1RM	60%AOP	Dynamic SQ
									Isometric KE & KF
	24		10		HL-RT:3–4 × 8	9(3)	75–80%1RM		
de Lemos Muller et al. [37]	24	Untrained	M13	BC, KE	BFR-RT:4 × 22	8(3)	30%1RM	110-150mmHg	Dynamic BC & KE
	25		M13		HL-RT:4 × 8	8(3)	80%1RM		
Ellefsen et al. [17]	23	Untrained	M12	KE	BFR-RT:5 × failure	12(2)	30%1RM	90-100mmHg	Dynamic KE
	23		M12		HL-RT:3 × 6–10	12(2)	75–90%1RM		
Fernandes et al. [38]	20	Untrained	F14	GS	BFR-RT:3 × 15–25	4(3)	45%1RM	160mmHg	Isometric GS
	20		F14		HL-RT:3 × 8–12	4(3)	75%1RM		
Jessee et al. [39]	21	Untrained	10	KE	BFR-RT1:4 × failure	8(2)	15%1RM	40%AOP	Dynamic KE
									Isometric KE
									Isokinetic KE (60°/s, 180°/s)
	21		10		BFR-RT2:4 × failure	8(2)	15%1RM	80%AOP	
	21		10		HL-RT:4 × failure	8(2)	70%1RM		
Karabulut et al. [9]	57	Untrained	M13	LP, KE	BFR-RT:30–15-15	6(3)	20%1RM	205mmHg	Dynamic LP & KE
	57		M13		HL-RT:3 × 8	6(3)	80%1RM		
Kim et al. [40]	26	Untrained	M10	LP, KE, KF	BFR-RT:2 × 10	3(3)	20%1RM	178 ± 20mmHg	Dynamic LP, KE & KF
	22		M10		HL-RT:2 × 10	3(3)	80%1RM		
Kim et al. [41]	21	Untrained	M9	BC	BFR-RT:30–15-15–15	8(3)	30%1RM	72 ± 11mmHg	Dynamic BC
									Isometric BC
	21		M9		HL-RT:3 × 10	8(3)	75%1RM		
Kim et al. [42]	63	Untrained	9	GS	BFR-RT:3 × failure	4(3)	20%1RM	160mmHg	Isometric GS
	63		10		HL-RT:3 × failure	4(3)	75%1RM		
Korkmaz et al. [43]	18	Trained	M11	KE	BFR-RT:30–15-15–15	6(2)	30%1RM	130-150mmHg	Isokinetic KE (60°/s, 80°/s)
	18		M12		HL-RT:4 × 12	6(2)	80%1RM		
Kubo et al. [10]	25	Untrained	M9	KE	BFR-RT:25–18-15–12	12(3)	20%1RM	180-240mmHg	Isometric KE
	25		M9		HL-RT:4 × 10	12(3)	80%1RM		
Laswati et al. [44]	33	Untrained	M6	BC	BFR-RT:30–15-15–15	5(2)	30%1RM	50mmHg	Isokinetic BC (60°/s)
	33		M6		HL-RT:3 × 12	5(2)	70%1RM		
Laurentino et al. [15]	20	Untrained	M10	KE	BFR-RT:3–4 × 15	8(2)	20%1RM	95 ± 10mmHg	Dynamic KE
	24		M9		HL-RT:3–4 × 8	8(2)	80%1RM		
Letieri et al. [45]	68	Untrained	F11	SQ, LP, KE, KF	BFR-RT1:30–15-15	16(3)	20–30%1RM	188 ± 5mmHg	Isokinetic KE & KF (60°/s)
	69		F10		BFR-RT2:30–15-15	16(3)	20–30%1RM	105 ± 7mmHg	
	67		F11		HL-RT:3–4 × 6–8	16(3)	70–80%1RM		
Libardi et al. [46]	64	Untrained	10	LP	BFR-RT:30–15-15–15	12(2)	20–30%1RM	67 ± 8mmHg	Dynamic LP
	65		8		HL-RT:4 × 10	12(2)	70–80%1RM		
Lixandrao et al. [47]	26	Untrained	M11	KE	BFR-RT1:2–3 × 15	12(2)	20%1RM	56 ± 8mmHg	Dynamic KE
	29		M14		BFR-RT2:2–3 × 15	12(2)	20%1RM	110 ± 9mmHg	
	26		M8		BFR-RT3:2–3 × 15	12(2)	40%1RM	55 ± 5mmHg	
	29		M10		BFR-RT4:2–3 × 15	12(2)	40%1RM	1055 ± 19mmHg	
	29		M9		HL-RT:2–3 × 10	12(2)	80%1RM		
Luebbers et al. [48]	16–17	Trained	M8	SQ	BFR-RT:30–15-15–15	6(2)	20%1RM		Dynamic SQ
	16–17		M9		HL-RT:3 × 10	6(2)	78%1RM		
Martin-Hernandez et al. [12]	20	Untrained	M10	KE	BFR-RT1:30–15-15–15	5(2)	20%1RM	110mmHg	Dynamic KE
									Isokinetic KE (60°/s, 80°/s)
	21		M10		BFR-RT2:2 × (30–15-15–15)	5(2)	20%1RM	110mmHg	
	21		M11		HL-RT:3 × 8	5(2)	85%1RM		
May et al. [49]	24	Untrained	M8	KE, KF	BFR-RT:30–15-15–15	7(3)	20%1RM	128mmHg	Dynamic KE & KF
	24		M9		HL-RT:4 × 8	7(3)	70%1RM		
Mendonca et al. [50]	22	Untrained	15	CR	BFR-RT:30–15-15–15	4(5)	20%1RM	60%AOP	Isometric CR
	22		15		HL-RT:4 × 10	4(5)	75%1RM		
Morley et al. [51]	24	Untrained	7	KE	BFR-RT1:3 × 10–12 + 1 × failure	8(3)	20%1RM	100%AOP	Dynamic KE
									Isometric KE
	21		6		BFR-RT2:3 × 10–12 + 1 × failure	8(3)	20%1RM	50%AOP	
	21		7		HL-RT:3 × 10–12 + 1 × failure	8(3)	70%1RM		
Ozaki et al. [16]	23	Untrained	M10	BP	BFR-RT:30–15-15–15	6(3)	30%1RM	100-160mmHg	Dynamic BP
	24		M9		HL-RT:3 × 10	6(3)	75%1RM		
Ramis et al. [52]	24	Untrained	M15	BC, KE	BFR-RT:4 × 23	8(3)	30%1RM	110-150mmHg	Isometric BC & KE
	25		M13		HL-RT:4 × 8	8(3)	80%1RM		Isokinetic BC & KE (60°/s)
Sharifi et al. [53]	21	Untrained	M8	LP, KE, KF, CP, BC	BFR-RT:9 × 20	6(3)	20–30%1RM	110-160mmHg	Dynamic LP & CP
	19		M8		HL-RT:9 × 10	6(3)	70–80%1RM		
	21		M8		BFR-RT:9 × 20	6(6)	20–30%1RM	110-160mmHg	
	19		M8		HL-RT:9 × 10	6(6)	70–80%1RM		
Shiromaru et al. [54]	23	Untrained	M15	KE	BFR-RT:3 × 15	3(4)	30%1RM	80%AOP	Dynamic KE
	23		M15		HL-RT:3 × 10	6(2)	80%1RM		
Sousa et al. [55]	24	Untrained	10	KE	BFR-RT:3 × failure	6(2)	30%1RM	142 ± 122mmHg	Isometric KE
	21		11		HL-RT:3 × failure	6(2)	80%1RM		
Sugiarto et al. [56]	26–45	Untrained	M6	BC	BFR-RT:30–15-15–15	5(2)	30%1RM	50mmHg	Isokinetic KE (60°/s, 120°/s, 180°/s)
	26–45		M6		HL-RT:3 × 12	5(2)	75%1RM		
Teixeira et al. [57]	24	Untrained	M8	KE	BFR-RT:3 × 15	8(2)	20%1RM	80%AOP	Dynamic KE
	24		M8		HL-RT:3 × 8	8(2)	70%1RM		
Thiebaud et al. [58]	59	Untrained	F6	CP, SR, SHP	BFR-RT:30–15-15	8(3)	10–30%1RM	80-120mmHg	Dynamic CP, SR & SHP
	62		F8		HL-RT:3 × 10	8(3)	70–90%1RM		
Vechin et al. [13]	62	Untrained	M8	LP	BFR-RT:30–15-15–15	12(2)	20–30%1RM	71 ± 9mmHg	Dynamic LP
	65		M8		HL-RT:4 × 10	12(2)	70–80%1RM		
Yasuda et al. [11]	22–32	Untrained	M10	BP, EE	BFR-RT:30–15-15–15	6(3)	30%1RM	100-160mmHg	Dynamic BP
									Isometric EE
	22–32		M10		HL-RT:3 × 10	6(3)	75%1RM		
Che et al. [19]	18	Trained	F8	SQ	BFR-RT:30–15-15–15	6(3)	30%1RM	180mmHg	Dynamic SQ
									Isokinetic KE & KF (60°/s, 180°/s)
	17		F8		HL-RT:4 × 8–10	6(3)	75%1RM		
Shanghua et al. [59]	21	Trained	M12	LP	BFR-RT:5 × 12	8(3)	40%1RM	200mmHg	Isokinetic KE & KF (60°/s, 180°/s)
	22		M12		HL-RT:5 × 12	8(3)	70%1RM		
Li et al. [18]	22	Trained	M8	SQ, SSQ, DF	BFR-RT:30–15-15–15	4(3)	30%1RM	200mmHg	Dynamic SQ
									Isokinetic KE & KF (60°/s)
	22		M8		HL-RT:4 × 8–12	4(3)	70%1RM		
Li et al. [60]	20	Trained	M10	BP, SQ	BFR-RT:30–15-15–15	6(2)	30%1RM	160-200mmHg	Dynamic SQ & BP
	20		M10		HL-RT:4 × 12	6(2)	70%1RM		
Wang et al. [61]	24	Trained	M9	SQ, SSQ, DF	BFR-RT:4 × 20–30	8(3)	30%1RM	200-220mmHg	Isokinetic KE & KF (60°/s)
	24		M9		HL-RT:4 × 12	8(3)	70%1RM		
Zhang et al. [62]	23	Trained	M8	SQ	BFR-RT1:5 × 10	5(2)	20%1RM	252mmHg	Dynamic SQ
	24		M8		BFR-RT2:5 × 11	5(2)	40%1RM	252mmHg	
	23		M8		HL-RT:5 × 12	5(2)	75%1RM		
Centner et al. [65]	28	Untrained	M14	CR	BFR-RT:30–15-15–15	14(3)	20–35%1RM	50%AOP	Dynamic CR
	28		M15		HL-RT:3 × 6–12	14(30	70–80%1RM		
De Araujo et al. [66]	23	Untrained	M10	BC	BFR-RT:30–15-15–15	6(2)	30%1RM	50%AOP	Dynamic BC
	23		M10		HL-RT:3 × 10–12	6(2)	70%1RM		
Horiuchi et al. [67]	18–30	Untrained	M12	LP, KE	BFR-RT:4 × 20	4(4)	30%1RM	130%AOP	Dynamic LP & KE
	18–30		M12		HL-RT:3 × 10	4(4)	75%1RM		
Judd et al. [68]	20	Trained	M4	BC	BFR-RT: 4 × 5	6(2)	30%1RM	40%AOP	Dynamic BC
	20		M4		HL-RT: 4 × 5	6(2)	80%1RM		
	20		F4		BFR-RT: 4 × 5	3(2)	30%1RM	40%AOP	
	20		F5		HL-RT: 4 × 5	3(2)	80%1RM		
Reece et al. [69]	21	Untrained	15	KE	BFR-RT: 3 × failure	6(3)	30%1RM	50%AOP	Dynamic KE
	22		15		HL-RT:3 × failure	6(3)	80%1RM		
Sousa-Silva et al. [70]	21	Untrained	M9	BC	BFR-RT:1 × 30 + 2–3 × 15	8(2)	30%1RM	50%AOP	Dynamic BC
	21		M9		HL-RT:3–4 × 10–12	8(2)	70%1RM		
Wang et al. [71]	20	Trained	M8	SQ	BFR-RT: 30–15-15–15	4(3)	30%1RM	200mmHg	Dynamic SQ
	20		M8		HL-RT;4 × 8–12	4(3)	70%1RM		

*M* male, *F* female

*BC* biceps curls, *BP* bench press, *CP* chest press, *DF* deadlift, *EE* elbow extension, *GS* grip strength, *KE* knee extension, *KF* knee flexion, *LP* leg press, *SHP* seated shoulder press, *CR* calf raises, *SQ* squat, *SSQ* split squat, *SR* Seated Rowing

**Table 2:** Characteristics of studies included in the meta-analysis of muscle hypertrophy adaptations

References	Age	Training Status	N	Exercise	Protocol	Weeks (times/wk)	Exercise load	Occlusion pressure	Muscle mass assessment		
									Method	Muscle groups	Test site
Bjornsen et al. [26]	24	Trained	9	SQ	BFR-RT:30–15-12–8	2(5)	24–30%1RM	120 mmHg	US	Rectus femoris	Distal
										Vastus lateralis	
										Vastus medialis	
										Vastus intermedius	
	26		8		HL-RT: 6–7 × 1–6	2(5)	74–76%1RM				
Buckner et al. [32]	18–35	Untrained	20	BC	BFR-RT1: 4 × failure	8(2)	15%1RM	57 mmHg	US	Upper arm	Mid
											Distal
	18–35		20		BFR-RT2: 4 × failure	8(2)	15%1RM	110 mmHg			
	18–35		20		HL-RT: 4 × failure	8(2)	70%1RM				
Centner et al. [33]	27	Untrained	M11	CR	BFR-RT:30–15-15–15	14(3)	20–35%1RM	50%AOP	US	Gastrocnemius	
	26		M14		HL-RT:3 × 6–12	14(3)	70–80%1RM				
Centner et al. [28]	28	Untrained	M14	LP,KE,CR	BFR-RT:30–15-15–15	14(3)	20–35%1RM	50%AOP	MRI	Rectus femoris,	Proximal Mid
											Distal
	27		M15		HL-RT:3 × 6–12	14(3)	70–85%1RM				
Cook et al. [34]	76	Untrained	12	KE,KF,LP	BFR-RT: ~ 3 × 30	12(2)	30%1RM	184 ± 25 mmHg	MRI	Quadriceps	
	77		12		HL-RT: ~ 3 × 15	12(2)	70%1RM				
Cook et al. [35]	18–22	Untrained	6	KE,LP	BFR-RT:2 × 25, 1 × failure	6(3)	20%1RM	180-200 mmHg	MRI	Quadriceps	
	18–22		6		HL-RT:2 × 10, 1 × failure	6(3)	70%1RM				
Davids et al. [36]	24	Trained	11	SQ,LP,KE,SSQ	BFR-RT:30 + 2–3 × 15	9(3)	30–40%1RM	60%AOP	MRI	Quadriceps	
	24		10		HL-RT:3–4 × 8	9(3)	75–80%1RM				
Ellefsen et al. [18]	23	Untrained	M12	KE	BFR-RT: 5 × failure	12(2)	30%1RM	90-100 mmHg	MRI	Quadriceps	Proximal
											Distal
	23		M12		HL-RT:3 × 6–10	12(2)	75–90%1RM				
Jessee et al. [41]	21	Untrained	10	KE	BFR-RT1: 4 × failure	8(2)	15%1RM	40%AOP	US	Quadriceps	Proximal
											Mid
											Distal
	21		10		BFR-RT2: 4 × failure	8(2)	15%1RM	80%AOP			
	21		10		HL-RT: 4 × failure	8(2)	70%1RM				
Kataoka et al. [65]	23	Untrained	27	CR	BFR-RT:4 × 16–39	6(3)	30%1RM	40%AOP	US	Gastrocnemius	
	23		27		HL-RT:4 × 12–19	6(3)	70%1RM			Soleus	
Kim et al. [66]	26	Untrained	M10	LP,KE,KF	BFR-RT:2 × 10	3(3)	20%1RM	178 ± 20 mmHg	CT	Thigh	Mid
	22		M10		HL-RT:2 × 10	3(3)	80%1RM				
Kim et al. [41]	21	Untrained	M9	BC	BFR-RT:30–15-15–15	8(3)	30%1RM	72 ± 11 mmHg	US	Upper arm	Mid
											Distal
	21		M9		HL-RT:3 × 10	8(3)	75%1RM				
Korkmaz et al. [43]	18	Trained	M11	KE	BFR-RT:30–15-15–15	6(2)	30%1RM	130-150 mmHg	US	Rectus femoris	Mid
										Vastus lateralis	
	18		M12		HL-RT:4 × 12	6(2)	80%1RM				
Kubo et al. [10]	25	Untrained	M9	KE	BFR-RT:25–18-15–12	12(3)	20%1RM	180-240 mmHg	MRI	Quadriceps	
	25		M9		HL-RT:4 × 10	12(3)	80%1RM				
Laurentino et al. [15]	20	Untrained	M10	KE	BFR-RT:3–4 × 15	8(2)	20%1RM	95 ± 10 mmHg	MRI	Quadriceps	Mid
	23		M9		HL-RT:3–4 × 8	8(2)	80%1RM				
Libardi et al. [46]	64	Untrained	10	LP	BFR-RT:30–15-15–15	12(2)	20–30%1RM	67 ± 8 mmHg	MRI	Quadriceps	Mid
	65		8		HL-RT:4 × 10	12(2)	70–80%1RM				
Lixandrao et al. [47]	26	Untrained	M11	KE	BFR-RT1:2–3 × 15	12(2)	20%1RM	55.5 ± 8 mmHg	MRI	Quadriceps	Mid
	28		M14		BFR-RT2:2–3 × 15	12(2)	20%1RM	109 ± 9 mmHg			
	26		M8		BFR-RT3:2–3 × 15	12(2)	40%1RM	54 ± 5 mmHg			
	28		M10		BFR-RT4:2–3 × 15	12(2)	40%1RM	105 ± 19 mmHg			
	29		M9		HL-RT:2–3 × 10	12(2)	80%1RM				
Martin-Hernandez et al. [12]	20	Untrained	M10	KE	BFR-RT1:30–15-15–15	5(2)	20%1RM	110 mmHg	US	Rectus femoris	Mid
										Vastus lateralis	
	21		M10		BFR-RT2:2 × (30–15-15–15)	5(2)	20%1RM	110 mmHg			
	20		M11		HL-RT:3 × 8	5(2)	85%1RM				
May et al. [49]	24	Untrained	M8	KE,KF	BFR-RT:30–15-15–15	7(3)	20%1RM	128 mmHg	CT	Quadriceps	Mid
											Distal
										Hamstrings	
	24		M9		HL-RT:4 × 8	7(3)	70%1RM				
Mendonca et al. [50]	22	Untrained	15	CR	BFR-RT:30–15-15–15	4(5)	20%1RM	60%AOP	US	Soleus	
	21		15		HL-RT:4 × 10	4(5)	75%1RM				
Ozaki et al. [16]	23	Untrained	M10	BP	BFR-RT:30–15-15–15	6(3)	30%1RM	100-160 mmHg	MRI	Triceps brachii	
										Pectoralis major	
	24		M9		HL-RT:3 × 10	6(3)	75%1RM				
Ramis et al. [52]	23	Untrained	M15	BC,KE	BFR-RT:4 × 23	8(3)	30%1RM	110-150 mmHg	US	Biceps brachii	
	24		M13		HL-RT:4 × 8	8(3)	80%1RM			Quadriceps	
Shiromaru et al. [54]	22	Untrained	M15	KE	BFR-RT:3 × 15	3(4)	30%1RM	80%AOP	MRI	Quadriceps	Mid
	22		M15		HL-RT:3 × 10	6(2)	80%1RM				
Teixeira et al. [57]	24	Untrained	M8	KE	BFR-RT:3 × 15	8(2)	20%1RM	80%AOP	MRI	Quadriceps	Mid
	24		M8		HL-RT:3 × 8	8(2)	70%1RM				
Thiebaud et al. [58]	59	Untrained	F6	CP,SR,SHP	BFR-RT:30–15-15	8(3)	10–30%1RM	80-120 mmHg	US	Biceps brachii	Distal
										Triceps brachii	
	62		F8		HL-RT:3 × 10	8(3)	70–90%1RM				
Vechin et al. [13]	62	Untrained	M8	LP	BFR-RT:30–15-15–15	12(2)	20–30%1RM	71 ± 9 mmHg	MRI	Quadriceps	Mid
	65		M8		HL-RT:4 × 10	12(2)	70–80%1RM				
Yasuda et al. [11]	22–32	Untrained	M10	BP,EE	BFR-RT:30–15-15–15	6(3)	30%1RM	100-160 mmHg	MRI	Triceps brachii	Mid
	22–32		M10		HL-RT:3 × 10	6(3)	75%1RM				
Sousa-Silva et al. [70]	21	Untrained	M9	BC	BFR-RT: 1 × 30 + 2–3 × 15	8(2)	30%1RM	50%AOP	US	Biceps brachii	Distal
	21		M9	BC	HL-RT:3–4 × 10–12	8(2)	70%1RM				

*M* male, *F* female

*1RM* one-repetition maximum, *AOP* arterial occlusion pressure, *CT* Computed Tomography, *MRI* magnetic resonance imaging, *US* ultrasonography,

*Distal* > 50% of the thigh or upper arm length, *Mid* = 50% of the thigh or upper arm length, *Proximal* < 50% of the thigh or upper arm length

*BC* biceps curls, *BP* bench press, *CP* chest press, *DF* deadlift, *EE* elbow extension, *GS* grip strength, *KE* knee extension, *KF* knee flexion, *LP* leg press, *SHP* seated shoulder press, *CR* calf raises, *SQ* squat, *SSQ* split squat, *SR* Seated Rowing

### Statistical Analyses

All analyses were performed using Comprehensive Meta-Analysis software (version 3.3, Biostat, Inc., Englewood, NJ, USA). The comparisons (BFR-RT vs. HL-RT) were calculated as the effect size difference (ES_diff_) using the difference in pre- and post- intervention mean and standard deviation values of muscle strength and mass, sample size and correlation between pre- and post-test for all groups. If the studies included in the meta-analysis did not report correlation between pre- and post-test, the following formula was used for estimation [20, 72]:

$$r = \frac{{S_{pre}^{2} + S_{post}^{2} - SD^{2} }}{{2 \times S_{pre} \times S_{post} }}$$

where S_pre_ and S_post_ are the standard deviation of pre-test and post-test, respectively. SD is the standard deviation of difference between pre- and post-test calculated using the following formula [1, 20]:

$$SD = \sqrt {\frac{{S_{pre}^{2} }}{n} + \frac{{S_{post}^{2} }}{n}}$$

Using the correction factor to correct the small sample size bias of all ES_diff_ [20, 31]. The correction factor is given by:

$${\text{Correction factor}} = 1 - \frac{3}{{4 \times \left( {n_{1} + n_{2} - 2} \right) - 1}}$$

The subjects of the studies included in the present meta-analysis came from different populations. Moreover, different training protocols and various strength and hypertrophy measurements and variables were utilized in these studies. All factors may have an impact on the effect of the intervention. Thus, the random-effects model was used to perform the meta-analysis [73]. The *I*^2^ statistics was used to assess heterogeneity. *I*^2^ values of 25%, 50% and 75% were set as low, moderate and high levels of heterogeneity, respectively. [74].

The first step was to compare the effects of BFR-RT and HL-RT on muscle strength and muscle mass. Subsequently, subgroup analyses were conducted to examine the effects of training status (trained vs. untrained individuals), sex, age, upper and lower limbs, test specificity (i.e. 1RM test vs. isometric or isokinetic tests) and region-specific adaptations of muscle hypertrophy. Based on the average age reported by the included studies, the age subgroups were divided into young (≤ 33 year) and old (≥ 57 year). Finally, according to the measured position reported by the studies, the results for muscle hypertrophy were categorized into three subgroups: proximal, middle and distal, which were < 50%, = 50% and > 50% of the length of the femur or humerus, respectively.

To identify the presence of highly influential studies that might bias the analyses, a sensitivity analysis was performed. The analysis was therefore conducted by removing one study at a time and then examining its effect on comparisons. If removal changed the significance level of ES_diff_ (i.e., from *P* ≤ 0.05 to *P* > 0.05, or vice versa), the study was considered as influential. This method has been used elsewhere [75]. The funnel plot, and Begg and Egger’s test were used to consider and assess publication bias, respectively. All data are presented as mean ± standard error. The significance level was set at *P* ≤ 0.05.

## Results

**Fig. 1:**
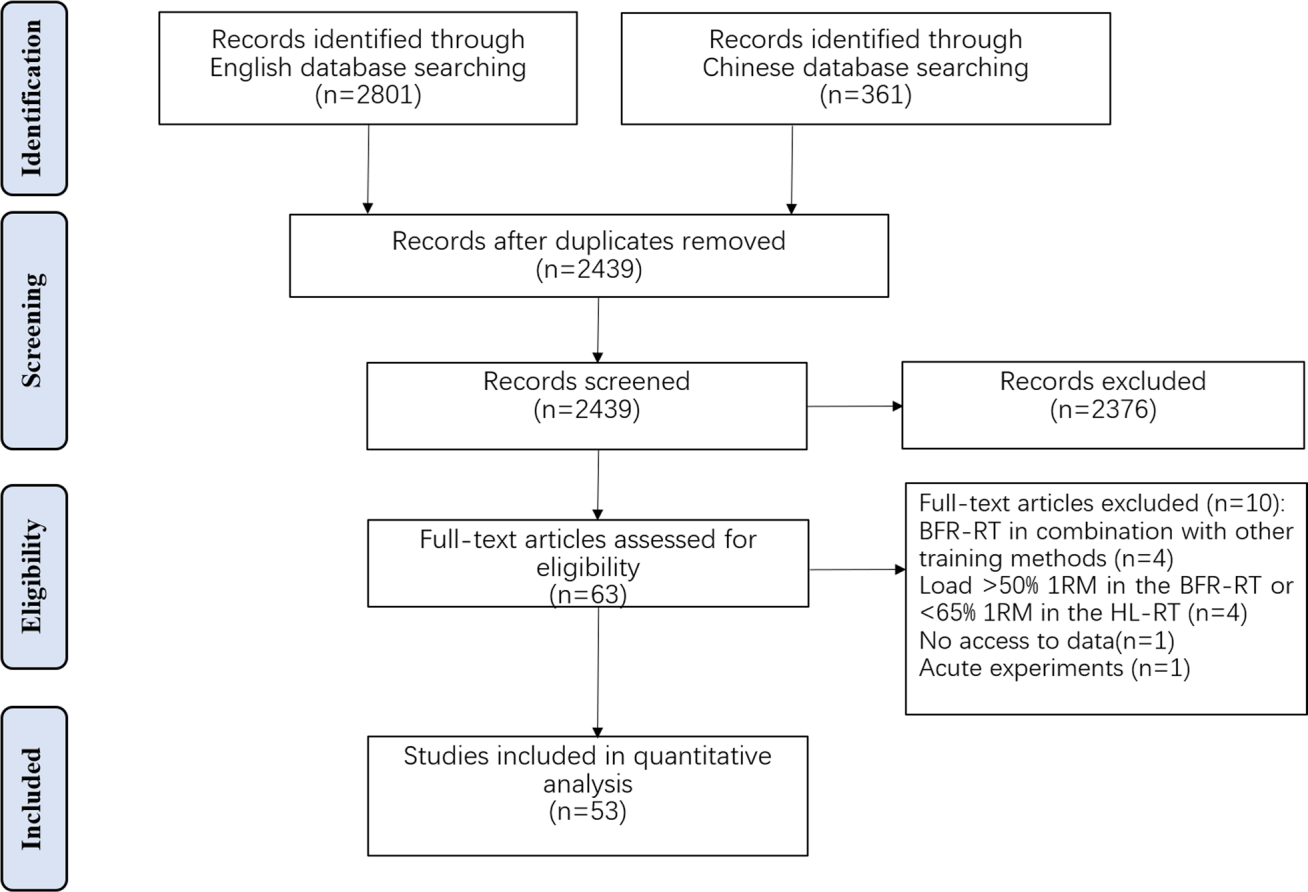
Flow diagram of the search and review process

The initial search retrieved 2801 English studies and Chinese 361studies. Afterwards, 723 duplicated studies were excluded. After evaluation of titles and abstracts, 2376 studies were removed, while the remaining 63 studies were assessed through full texts. Finally, 53 studies were considered to meet the inclusion criteria (Fig. 1), 51 of which were included in the muscle strength analysis (Table 1) and 28 in the muscle hypertrophy analysis (Table 2). In addition, by contacting the authors, the muscle hypertrophy data of one study were obtained [28]. However, after multiple attempts to contact the author, the muscle strength and hypertrophy data for another study were not included as the author did not respond [76].

### Muscle Strength

**Fig. 2:**
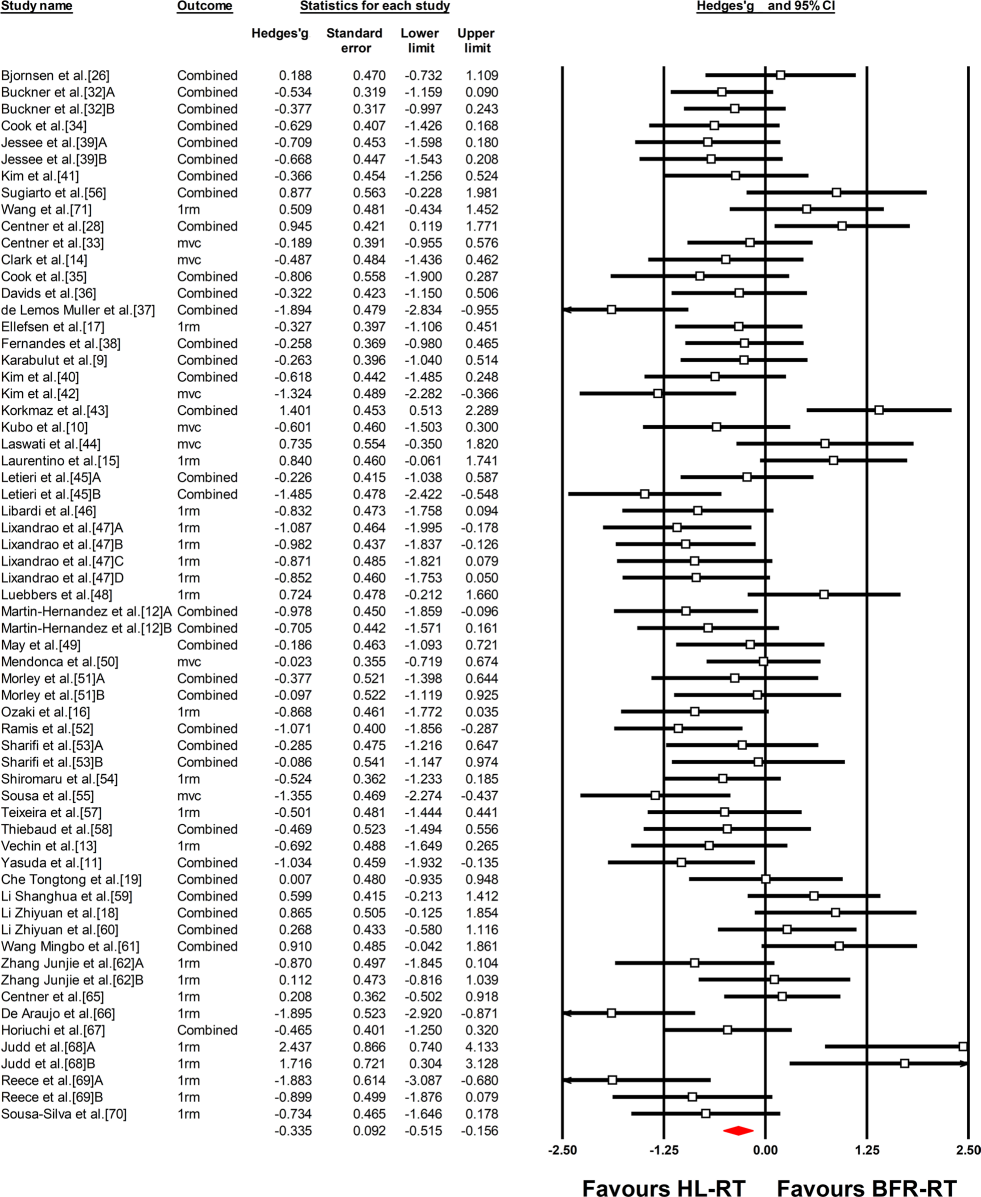
Forest plot of the effect size difference between BFR-RT versus HL-RT for muscle strength. The different capital letters (i.e. A, B, C, D) after the reference number are used to represent different training protocols for the same study. Hedges’g represents effect size difference. Red diamond represents overall Hedges’g. *1rm* 1RM test, *BFR-RT* blood-flow restriction low-load resistance training, *CI* confidence interval, *Combined* mean of multiple outcomes from the same training protocol, *HL-RT* high-load resistance training, *mvc* isometric or isokinetic tests

Fifty-one studies involving 1164 participants were included in the present meta-analysis to compare muscle strength gains. In the studies that investigated the trained population, only one study adopted 9 weeks of training duration and one study adopted a training frequency of 5 sessions per week (Fig. 2).

**Fig. 3:**
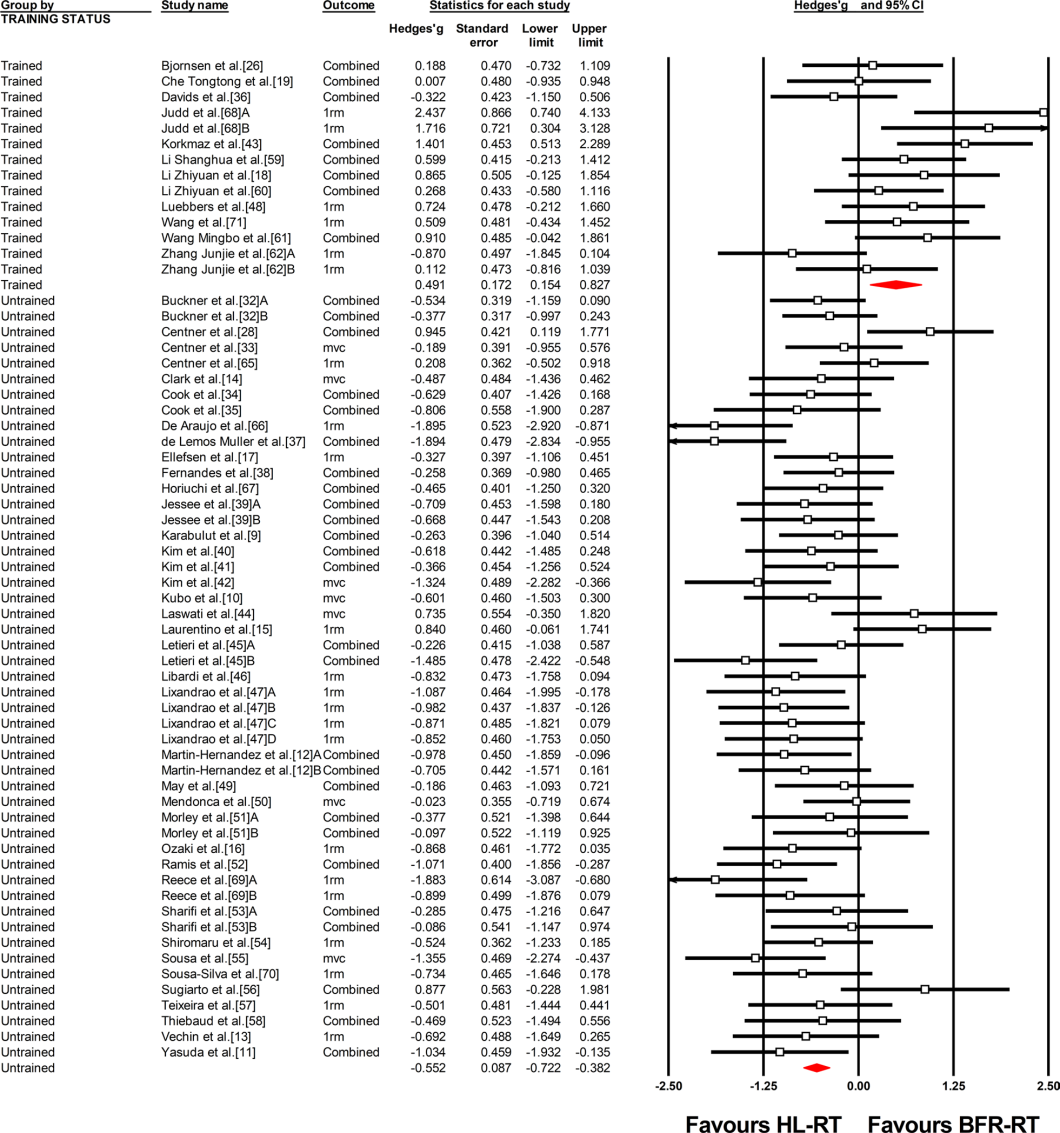
Forest plot of the effect size difference between BFR-RT versus HL-RT for muscle strength according to training status. The different capital letters (i.e. A, B, C, D) after the reference number are used to represent different training protocols for the same study. Hedges’g represents effect size difference. Red diamonds represent overall Hedges’g of subgroups. *1rm* 1RM test, *BFR-RT* blood-flow restriction low-load resistance training, *CI* confidence interval, *Combined* mean of multiple outcomes from the same training protocol, *HL-RT* high-load resistance training, *mvc* isometric or isokinetic tests

The overall ES_diff_ demonstrated significantly lower gains in muscle strength for BFR-RT compared with HL-RT (ES_diff_ = − 0.335 ± 0.092, 95% confidence interval [CI] − 0.515 to − 0.156) (Fig. 2 and Table 3). However, when considering training status, the differences between trained and untrained subgroups were significant (Q = 29.39, *P* < 0.01) (Table 3). Significantly higher strength gains for BFR-RT were observed compared with HL-RT in the trained group (ES_diff_ = 0.491 ± 0.172, 95% CI 0.154 to 0.827) (Fig. 3 and Table 3). In contrast, the strength gains of HL-RT were significantly higher than that of BFR-RT in the untrained group (ES_diff_ = − 0.552 ± 0.087, 95%CL − 0.722 to − 0.382) (Fig. 3 and Table 3). In trained individuals, there were no significant differences between the different sexes, limbs, durations, frequency and test types (Table 3 and Additional file 1: Figs. S1–S5). In untrained individuals, there were also no significant differences between the different sexes, age, limbs, training duration and frequency (Table 3 and Additional file 1: Figs. S6–S11).

**Table 3:** Summary of meta-analysis results for muscle strength

	Subgroups	N	ES_diff_	Standard error	95% Confidence interval		*P* value	Between group		Heterogeneity		
					Lower limit	Upper limit		Q-value	*P* value	Q-value	*P* value	*I*^*2*^
Overall effect												
		63	− 0.335	0.092	− 0.515	− 0.156	< 0.01			157.542	< 0.01	60.65
Training status												
	Trained	14	0.491	0.172	0.154	0.827	< 0.01	29.392	< 0.01	27.047	0.01	51.94
	Untrained	49	− 0.552	0.087	− 0.722	− 0.382	< 0.01			83.634	< 0.01	42.61
Trained												
Sex	Female	2	0.682	0.572	− 0.440	1.804	0.23	0.014	0.91	3.896	0.05	74.33
	Male	10	0.609	0.236	0.146	1.072	0.01			18.791	0.03	52.11
Limbs	Lower limb	12	0.391	0.198	0.003	0.779	0.05	1.495	0.22	18.374	0.07	40.13
	Upper limb	3	1.011	0.467	0.095	1.926	0.03			8.736	0.01	77.11
Duration	≤ 4wk	4	0.724	0.371	− 0.004	1.452	0.05	0.214	0.64	3.420	0.33	12.28
	= 5–8wk	9	0.520	0.238	0.053	0.987	0.03			19.563	0.01	59.11
Frequency	2/wk	7	0.660	0.300	0.072	1.248	0.03	0.330	0.57	20.471	< 0.01	70.69
	3/wk	6	0.415	0.303	− 0.179	1.010	0.17			5.821	0.32	14.10
Test type	Non-specific test	6	0.541	0.282	− 0.011	1.094	0.05	0.352	0.55	11.211	0.05	55.40
	Specific test	11	0.330	0.218	− 0.098	0.758	0.13			20.499	0.02	51.22
Untrained												
Sex	Female	5	− 0.638	0.297	− 1.220	− 0.055	0.03	0.143	0.71	5.529	0.24	27.66
	Male	31	− 0.516	0.120	− 0.751	− 0.282	< 0.01			68.109	< 0.01	55.95
Age	Old	8	− 0.714	0.205	− 1.115	− 0.312	< 0.01	0.573	0.45	7.159	0.41	2.22
	Young	40	− 0.544	0.091	− 0.721	− 0.367	< 0.01			69.180	< 0.01	43.63
Limbs	Lower limb	36	− 0.618	0.111	− 0.836	− 0.400	< 0.01	0.514	0.47	73.921	< 0.01	52.65
	Upper limb	17	− 0.477	0.162	− 0.794	− 0.160	< 0.01			38.871	< 0.01	58.84
Duration	≤ 4wk	7	− 0.493	0.216	− 0.916	− 0.070	0.02	0.278	0.87	5.107	0.53	0.00
	= 5–8wk	28	− 0.593	0.116	− 0.820	− 0.365	< 0.01			51.803	< 0.01	47.88
	≥ 9wk	14	− 0.507	0.158	− 0.816	− 0.198	< 0.01			25.833	0.02	49.68
Frequency	2/wk	21	− 0.596	0.131	− 0.853	− 0.339	< 0.01	0.795	0.67	35.971	0.02	44.40
	3/wk	25	− 0.560	0.121	− 0.797	− 0.323	< 0.01			44.477	0.01	46.04
	4/wk	2	− 0.231	0.388	− 0.991	0.528	0.55			0.683	0.41	0.00
Test specificity	Non-specific test	24	− 0.422	0.120	− 0.657	− 0.188	< 0.01	3.573	0.06	33.745	0.07	31.84
	Specific test	37	− 0.715	0.099	− 0.909	− 0.522	< 0.01			70.945	< 0.01	49.26

*ES*_*diff*_ effect size difference

The sensitivity analysis conducted by deleting one study at a time and re-analyzing the data showed that none of the studies had a significant impact on muscle strength results. Inspection of the funnel plots indicated no evidence of publication bias (Additional file 1: Fig. S12). The results of the Begg test showed that Kendall’s tau with continuity correction was equal to − 0.02 (*P* = 0.84), and Egger’s regression intercept was equal to 1.09 (*P* = 0.41).

### Muscle Hypertrophy

Twenty-eight studies involving 703 participants were included in the present meta-analysis to compare muscle hypertrophy gains. However, only three studies investigated the trained population. In addition, of the studies that investigated the untrained population, only one included female subjects, and only one adopted a training frequency of 4 sessions per week.

**Fig. 4:**
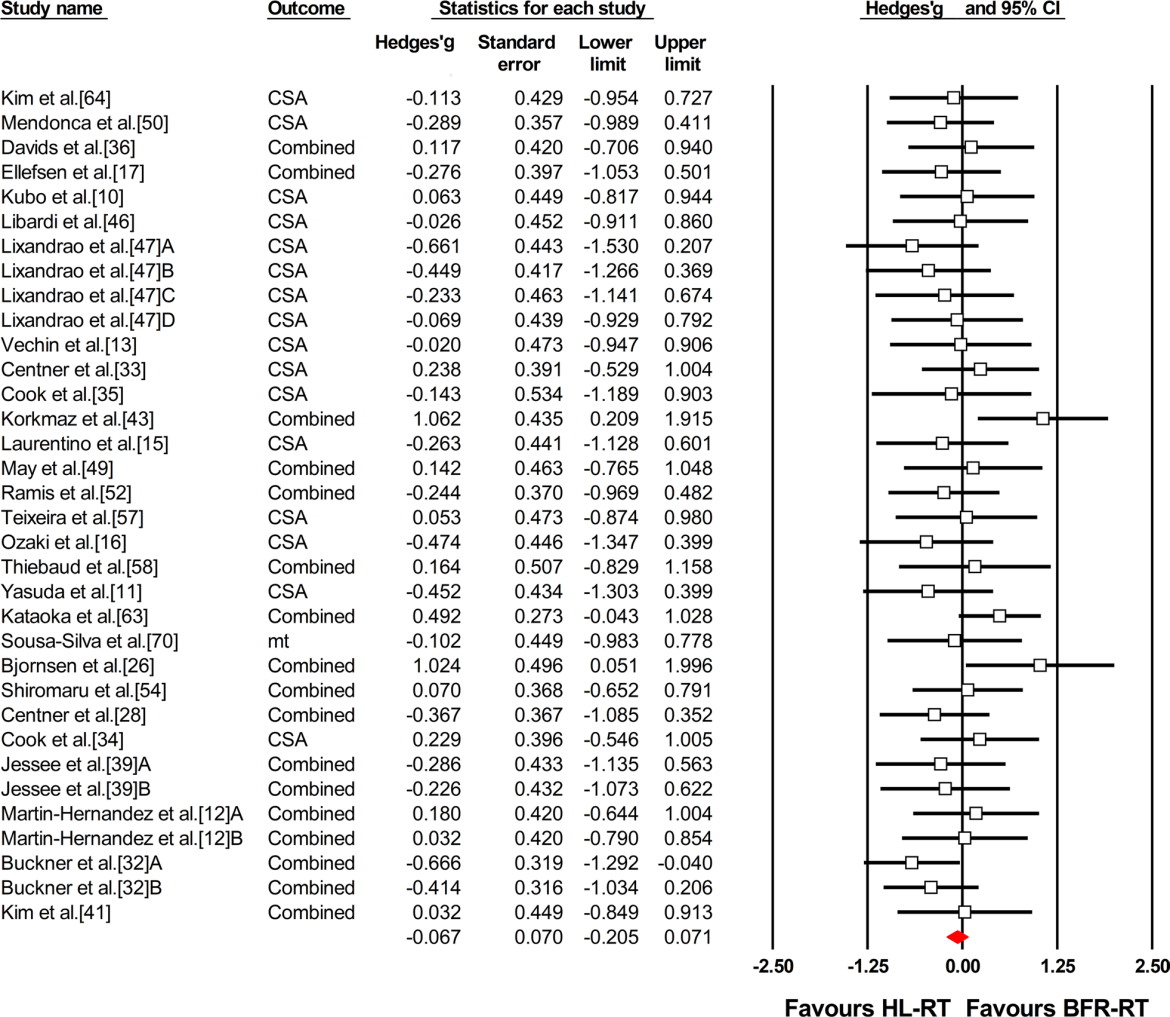
Forest plot of the effect size difference between BFR-RT versus HL-RT for muscle hypertrophy. The different capital letters (i.e. A, B, C, D) after the reference number are used to represent different training protocols for the same study. Hedges’g represents effect size difference. Red diamond represents overall Hedges’g. *BFR-RT* blood-flow restriction low-load resistance training, *CI* confidence interval, *Combined* mean of multiple outcomes from the same training protocol, *CSA* cross-sectional area, *HL-RT* high-load resistance training, *mt* muscle thickness

**Fig. 5:**
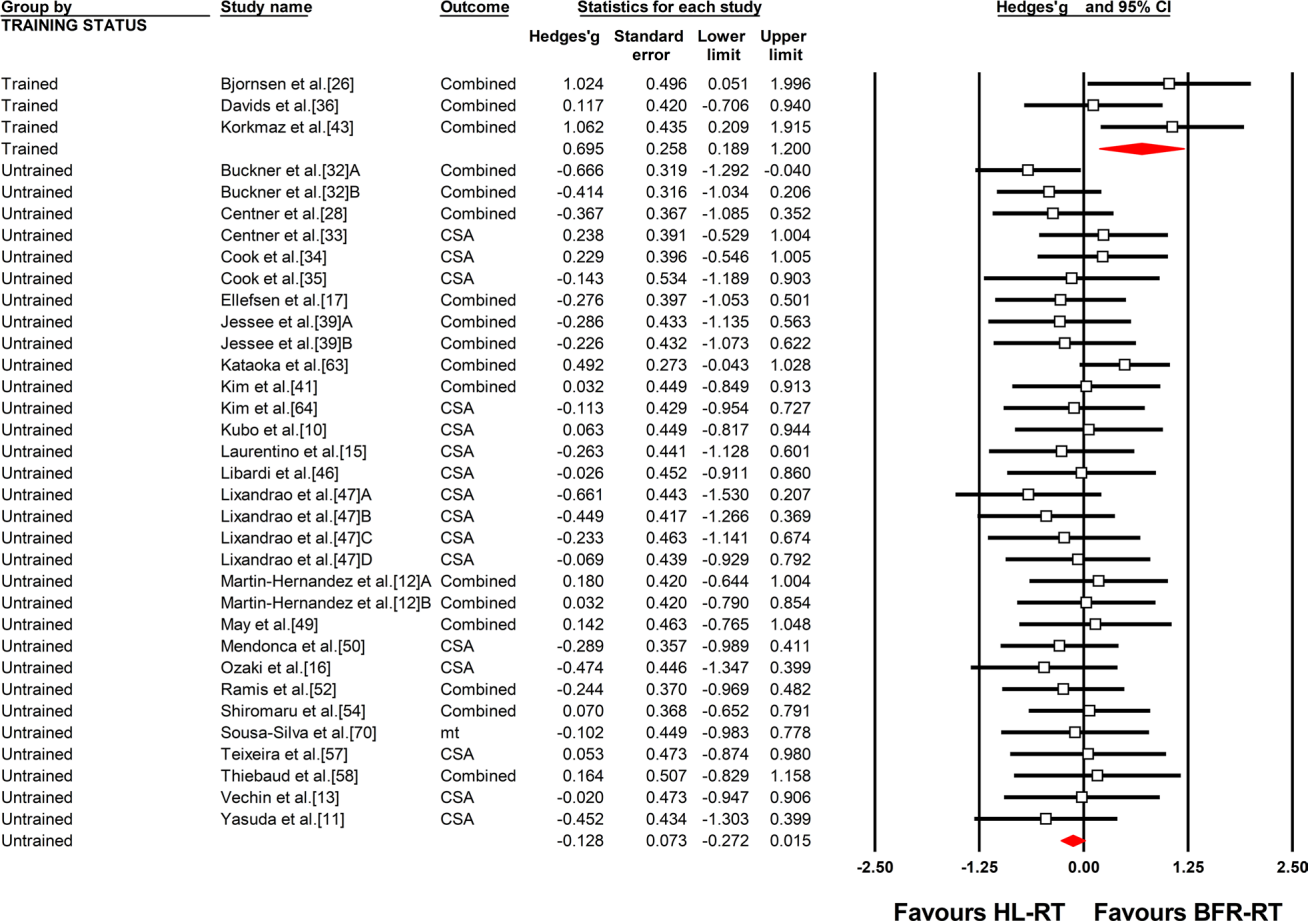
Forest plot of the effect size difference between BFR-RT versus HL-RT for muscle hypertrophy according to training status. The different capital letters (i.e. A, B, C, D) after the reference number are used to represent different training protocols for the same study. Hedges’g represents effect size difference. Red diamonds represent overall Hedges’g of subgroups. *BFR-RT* blood-flow restriction low-load resistance training, *CI* confidence interval, *Combined* mean of multiple outcomes from the same training protocol, *CSA* cross-sectional area, *HL-RT* high-load resistance training, *mt* muscle thickness

The overall ES_diff_ suggested similar gains in muscle mass between BFR-RT and HL-RT (ES_diff_ = − 0.067 ± 0.070, 95% CI − 0.205 to 0.071) (Fig. 4 and Table 4). However, when considering training status, the differences between trained and untrained subgroups were significant (Q = 9.41, *P* < 0.01) (Table 4). Significantly higher muscle hypertrophy gains for BFR-RT were observed compared with HL-RT in the trained subgroup (ES_diff_ = 0.695 ± 0.258, 95% CI 0.189–1.200). In contrast, the muscle mass gains with BFR-RT were similar to those with HL-RT in the untrained subgroup (ES_diff_ = − 0.128 ± 0.073, 95% CI − 0.272 to 0.015) (Fig. 5 and Table 4). However, in untrained individuals, there were no significant differences between the different age, limbs, duration and frequency, and region-specific adaptations in muscle mass (Additional file 1: Figs. S13–S18 and Table 4).

**Table 4:** Summary of meta-analysis results for muscle hypertrophy

		N	ES_diff_	Standard error	95% CI		Between Group			Heterogeneity		
					Lower limit	Upper limit	*P* value	Q-value	*P* value	Q-value	*P* value	*I*^*2*^*(%)*
Overall effect												
		34	− 0.067	0.070	− 0.205	0.071	0.34			29.578	0.64	0.00
Training status												
	Trained	3	0.695	0.258	0.189	1.200	0.01	9.413	< 0.01	3.048	0.22	34.39
	Untrained	31	− 0.128	0.073	− 0.272	0.015	0.08			17.116	0.97	0.00
Untrained												
Age	Old	4	0.096	0.226	− 0.346	0.538	0.67	1.106	0.29	0.265	0.97	0.00
	Young	27	− 0.155	0.077	− 0.306	− 0.003	0.05			15.746	0.94	0.00
Limbs	Lower limbs	24	− 0.053	0.084	− 0.217	0.111	0.52	3.398	0.07	10.961	0.98	0.00
	Upper limbs	8	− 0.354	0.140	− 0.628	− 0.080	0.01			3.231	0.86	0.00
Duration	≤ 4 wk	3	− 0.115	0.220	− 0.546	0.317	0.60	0.254	0.88	0.489	0.78	0.00
	= 5–8 wk	18	− 0.102	0.095	− 0.289	0.084	0.28			12.718	0.75	0.00
	≥ 9 wk	10	− 0.185	0.134	− 0.448	0.079	0.17			3.656	0.93	0.00
Frequency	2/wk	17	− 0.219	0.100	− 0.416	− 0.022	0.03	2.128	0.15	7.113	0.97	0.00
	3/wk	13	0.000	0.112	− 0.219	0.220	0.99			7.663	0.81	0.00
Assessment region (Thigh)	Distal	5	− 0.069	0.184	− 0.430	0.291	0.71	1.805	0.41	0.478	0.98	0.00
	Mid	16	− 0.149	0.107	− 0.359	0.062	0.17			4.550	0.99	0.00
	Proximal	4	− 0.417	0.203	− 0.816	− 0.018	0.04			0.470	0.93	0.00
Assessment region (Upper-arm)	Distal	5	− 0.242	0.172	− 0.579	0.094	0.16	1.762	0.18	1.912	0.75	0.00
	Mid	4	− 0.577	0.184	− 0.938	− 0.216	< 0.01			3.881	0.28	22.70

*2/wk* 2 sessions per week, *3/w* 3 sessions per week, *ES*_*diff*_ effect size difference

The sensitivity analysis showed that muscle hypertrophic adaptation was not affected by any particular study. Inspection of the funnel plots indicated no evidence of publication bias (Additional file 1: Fig. S19). The results of the Begg test show that Kendall’s tau with continuity correction was equal to 0.17 (*P* = 0.16), and Egger’s regression intercept was equal to 0.52 (*P* = 0.65).

## Discussion

The purpose of the current study was to compare the effects of BFR-RT and HL-RT on the muscle strength and hypertrophy, using HL-RT as a control to evaluate the effects and characteristics of BFR-RT. The main finding of the present study was that training status was an important influencing factor in the effects of BFR-RT. The trained individuals will get greater muscle strength and hypertrophy gains from BFR-RT as compared with HL-RT. However, in the untrained individuals, the results demonstrated that superior gains in muscle strength and similar muscle mass for HL-RT as compared with BFR-RT.

### Effect of BFR-RT on Trained Individuals

The analysis results of trained individuals (ES_diff_ = 0.491) suggested that in the comparison of these two training modalities, 69% of trained individuals may obtain greater gains in muscle strength with BFR-RT [77].

Training is initially characterized by neural adaptations, however, later as neural adaptations reach a plateau, muscular adaptation (i.e., hypertrophy) dominates [23, 78]. In intermediate and advanced training, the progress of strength training is limited to the degree of muscle adaptation that can be achieved [23]. Hakkinen et al. reported that during one-year traditional strength training, advanced weight-lifters show limited potential for further neural adaptations, and the total mean muscle fiber area did not increase significantly [79]. However, mounting research indicates that BFR-RT can promote muscle hypertrophy in athletes [18, 26, 43, 80, 81], and even in elite powerlifters the muscle fiber CSA increased more with BFR-RT than with HL-RT [26].

Metabolic stress was believed to be one of the factors promoting muscle hypertrophy [82]. Compared with other strength training protocols, BFR-RT was believed to produce a higher level of metabolic stress [83]. Studies suggested that compared with normoxic conditions, resistance training under the hypoxic condition caused greater metabolic and hormonal responses [84, 85], whereas it was believed that blood flow restriction could cause similar muscle hypoxia as compared with systemic hypoxia [86]. In this study, the results (Table 4) showed that in the trained individuals, superior muscle hypertrophy gains were observed for BFR-RT as compared with HL-RT. Put another way, 76% of the trained population may obtain greater gains in muscle hypertrophy with BFR-RT [77]. In fact, our results showed that the average relative strength change [(pre-training − post-training)/pre-training × 100] with BFR-RT (8.4% ± 1.09) was twice that of HL-RT (3.96% ± 0.66) in the trained individuals.

### Effect of BFR-RT on Untrained Individuals

Being different from the trained subjects, the analysis results for untrained individuals (ES_diff_ = − 0.552) suggested that about 70% of untrained individuals may experience greater gains in muscle strength with HL-RT [77].

However, previous studies have found that the muscle activation level of BFR-RT was higher than that of the same intensity (low load) resistance exercise [87–90], its muscle activation level is still low as compared with HL-RT [91–93]. For example, Cook et al. [92] reported that muscle activation level (according to surface electromyography) was greater in the HL-RT at the beginning and end of exercise compared with the BFR-RT. It has been suggested that increasing the occlusion pressure (from 40 to 60% occlusive pressure) could increase the activation level of muscle [94], but recent research showed that even with higher occlusive pressure (80%), the activation level of BFR-RT on muscle was also significantly lower than HL-RT [91]. While these findings were based on surface electromyography, BFR-RT may not achieve the same level of muscle activation and produce the same neural stimulation as HL-RT [83, 95].

### Limitations

The current meta-analysis has some limitations. The lack of studies including females limited generalizability of the findings. Because of the sparse number of studies, the results comparing muscle hypertrophy in trained individuals should be interpreted with caution. In addition, the data from one study were not included [76]. However, the sensitivity analysis revealed that no single study had a significant impact on the analysis results. Therefore, the absence of these data would unlikely to have affected the current results and their interpretation.

## Conclusion

The present meta-analysis indicates that training status is an important factor influencing the effects of BFR-RT. Compared to HL-RT, trained individuals can obtain greater strength and hypertrophy gains from BFR-RT. However, in untrained individuals, the results demonstrate that superior muscle strength and similar mass gains for HL-RT.

From a practical standpoint, BFR-RT could be a beneficial supplemental training protocol for trained population. It has been demonstrated that the combination of BFR- and HL-RT was more beneficial for the increase of muscle strength [97]. Thus, healthy individuals or athletes are likely to maximize their training adaptations by combining these two training methods [4, 98]. Finally, it is important to highlight that BFR-RT remains a valid and effective alternative for people who cannot perform high-load resistance training.

### Supplementary Information

**Additional file 1**

## Data Availability

The datasets analysed during the current study are available from the corresponding author on reasonable request.

## Abbreviations

1RM: One repetition maximum
BFR-RT: Low-load resistance training (< 50% 1RM) combined with blood flow restriction
CI: Confidence interval
CSA: Cross-sectional area
ES_diff_: Effect size difference
HL-RT: High-load resistance training
PEDro: Physiotherapy Evidence Database

## References

[1] Schoenfeld BJ, Wilson JM, Lowery RP, Krieger JW (2016). Muscular adaptations in low- versus high-load resistance training: a meta-analysis. Eur J Sport Sci.

[2] Kraemer WJ, Ratamess NA (2004). Fundamentals of resistance training: progression and exercise prescription. Med Sci Sports Exerc.

[3] Campos GER, Luecke TJ, Wendeln HK, Toma K, Hagerman FC, Murray TF (2002). Muscular adaptations in response to three different resistance-training regimens: specificity of repetition maximum training zones. Eur J Appl Physiol.

[4] Scott B, Loenneke J, Slattery K, Dascombe B (2015). Exercise with blood flow restriction: an updated evidence-based approach for enhanced muscular development. Sports Med.

[5] Pignanelli C, Petrick HL, Keyvani F, Heigenhauser GJF, Quadrilatero J, Holloway GP (2020). Low-load resistance training to task failure with and without blood flow restriction: muscular functional and structural adaptations. Am J Physiol Regul Integr Comp Physiol.

[6] Hughes L, Paton B, Rosenblatt B, Gissane C, Patterson SD (2017). Blood flow restriction training in clinical musculoskeletal rehabilitation: a systematic review and meta-analysis. Br J Sports Med.

[7] Slysz J, Stultz J, Burr JF (2016). The efficacy of blood flow restricted exercise: a systematic review & meta-analysis. J Sci Med Sport.

[8] Loenneke JP, Wilson JM, Marin PJ, Zourdos MC, Bemben MG (2012). Low intensity blood flow restriction training: a meta-analysis. Eur J Appl Physiol.

[9] Karabulut M, Abe T, Sato Y, Bemben MG (2010). The effects of low-intensity resistance training with vascular restriction on leg muscle strength in older men. Eur J Appl Physiol.

[10] Kubo K, Komuro T, Ishiguro N, Tsunoda N, Sato Y, Ishii N (2006). Effects of low-load resistance training with vascular occlusion on the mechanical properties of muscle and tendon. J Appl Biomech.

[11] Yasuda T, Ogasawara R, Sakamaki M, Ozaki H, Sato Y, Abe T (2011). Combined effects of low-intensity blood flow restriction training and high-intensity resistance training on muscle strength and size. Eur J Appl Physiol.

[12] Martin-Hernandez J, Marin PJ, Menendez H, Ferrero C, Loenneke JP, Herrero AJ (2013). Muscular adaptations after two different volumes of blood flow-restricted training. Scand J Med Sci SPORTS.

[13] Vechin FC, Libardi CA, Conceição MS, Damas FR, Lixandrão ME, Berton RPB (2015). Comparisons between low-intensity resistance training with blood flow restriction and high-intensity resistance training on quadriceps muscle mass and strength in elderly. J Strength Cond Res.

[14] Clark BC, Manini TM, Hoffman RL, Williams PS, Guiler MK, Knutson MJ (2011). Relative safety of 4 weeks of blood flow-restricted resistance exercise in young, healthy adults. Scand J Med Sci Sports.

[15] Laurentino GC, Ugrinowitsch C, Roschel H, Aoki MS, Soares AG, Neves M (2012). Strength training with blood flow restriction diminishes myostatin gene expression. Med Sci Sports Exerc.

[16] Ozaki H, Yasuda T, Ogasawara R, Sakamaki-Sunaga M, Naito H, Abe T (2013). Effects of high-intensity and blood flow-restricted low-intensity resistance training on carotid arterial compliance: role of blood pressure during training sessions. Eur J Appl Physiol.

[17] Ellefsen S, Hammarstrom D, Strand TA, Zacharoff E, Whist JE, Rauk I (2015). Blood flow-restricted strength training displays high functional and biological efficacy in women: a within-subject comparison with high-load strength training. Am J Physiol Regul Integr Comp Physiol.

[18] Li Z, Zhao Z, Wang M, Chen C, Wei W, Liang Y (2019). Effect of 4 weeks KAATSU training on body composition and maximum strength of male handball players. China Sport Sci Technol.

[19] Che T, Li Z, Yang T, Chen Z, Wang S (2022). Effects of six-week low intensity KAATSU training combined with high intensity resistance training on core area and lower limb muscle strength in adolescent female wrestlers. J Cap Univ Phys Educ Sports.

[20] Lixandrão M, Ugrinowitsch C, Berton R, Vechin F, Conceição M, Damas F (2018). Magnitude of muscle strength and mass adaptations between high-load resistance training versus low-load resistance training associated with blood-flow restriction: a systematic review and meta-analysis. Sports Med.

[21] Grønfeldt BM, Lindberg Nielsen J, Mieritz RM, Lund H, Aagaard P (2020). Effect of blood-flow restricted vs heavy-load strength training on muscle strength: systematic review and meta-analysis. Scand J Med Sci Sports.

[22] Farrell PA, Joyner MJ, Caiozzo VJ. In: American College of Sports Medicine, editors. ACSM’s advanced exercise physiology. 2nd ed. Philadelphia: Wolters Kluwer Health/Lippincott Williams & Wilkins; 2012.

[23] Sale DG, Komi PV (2003). Neural adaptation to strength training. Strength power sport.

[24] Vissing K, Groennebaek T, Wernbom M, Aagaard P, Raastad T (2020). Myocellular adaptations to low-load blood flow restricted resistance training. Exerc Sport Sci Rev.

[25] Hill EC, Housh TJ, Keller JL, Smith CM, Anders JV, Schmidt RJ (2021). Patterns of responses and time-course of changes in muscle size and strength during low-load blood flow restriction resistance training in women. Eur J Appl Physiol.

[26] Bjornsen T, Wernbom M, Kirketeig A, Paulsen G, Samnoy L, Baekken L (2019). Type 1 muscle fiber hypertrophy after blood flow-restricted training in powerlifters. Med Sci Sports Exerc.

[27] Kacin A, Strazar K (2011). Frequent low-load ischemic resistance exercise to failure enhances muscle oxygen delivery and endurance capacity. Scand J Med Sci Sports.

[28] Centner C, Jerger S, Lauber B, Seynnes O, Friedrich T, Lolli D (2022). Low-load blood flow restriction and high-load resistance training induce comparable changes in patellar tendon properties. Med Sci Sports Exerc.

[29] Buckner SL, Jessee MB, Mattocks KT, Mouser JG, Counts BR, Dankel SJ (2017). Determining strength: a case for multiple methods of measurement. Sports Med.

[30] Verhagen AP, de Vet HCW, de Bie RA, Kessels AGH, Boers M, Bouter LM (1998). The delphi list: a criteria list for quality assessment of randomized clinical trials for conducting systematic reviews developed by Delphi consensus. J Clin Epidemiol.

[31] Borenstein M, Hedges LV, Higgins JPT, Rothstein HR (2009). Introduction to meta-analysis.

[32] Buckner SL, Jessee MB, Dankel SJ, Mattocks KT, Mouser JG, Bell ZW (2020). Blood flow restriction does not augment low force contractions taken to or near task failure. Eur J Sport Sci.

[33] Centner C, Lauber B, Seynnes OR, Jerger S, Sohnius T, Gollhofer A (2019). Low-load blood flow restriction training induces similar morphological and mechanical Achilles tendon adaptations compared with high-load resistance training. J Appl Physiol.

[34] Cook SB, LaRoche DP, Villa MR, Barile H, Manini TM (2017). Blood flow restricted resistance training in older adults at risk of mobility limitations. Exp Gerontol.

[35] Cook SB, Scott BR, Hayes KL, Murphy BG (2018). Neuromuscular adaptations to low-load blood flow restricted resistance training. J Sports Sci Med.

[36] Davids CJ, Naess TC, Moen M, Cumming KT, Horwath O, Psilander N (2021). Acute cellular and molecular responses and chronic adaptations to low-load blood flow restriction and high-load resistance exercise in trained individuals. J Appl Physiol.

[37] de Lemos Muller CH, Ramis TR, Ribeiro JL (2019). Effects of low-load resistance training with blood flow restriction on the perceived exertion, muscular resistance and endurance in healthy young adults. Sport Sci Health.

[38] Zanardini Fernandes D, Müller Reis Weber V, Amaral da Silva MP, de Lima Stavinski NG, Campos de Oliveira LE, Casoto Tracz EH (2020). Effects of blood flow restriction training on handgrip strength and muscular volume of young women. Int J Sports Phys Ther.

[39] Jessee MB, Buckner SL, Mouser JG, Mattocks KT, Dankel SJ, Abe T (2018). Muscle adaptations to high-load training and very low-load training with and without blood flow restriction. Front Physiol.

[40] Kim SJ, Sherk VD, Bemben MG, Bemben DA (2009). Effects of short-term, low-intensity resistance training with vascular restriction on arterial compliance in untrained young men. Int J KAATSU Train Res.

[41] Kim D, Loenneke JP, Ye X, Bemben DA, Beck TW, Larson RD (2017). Low-load resistance training with low relative pressure produces muscular changes similar to high-load resistance training. Muscle Nerve.

[42] Kim J, Lang JA, Pilania N, Franke WD (2017). Effects of blood flow restricted exercise training on muscular strength and blood flow in older adults. Exp Gerontol.

[43] Korkmaz E, Donmez G, Uzuner K, Babayeva N, Torgutalp SS, Ozcakar L (2022). Effects of blood flow restriction training on muscle strength and architecture. J Strength Cond Res.

[44] Laswati H, Sugiarto D, Poerwandari D, Pangkahila JA, Kimura H (2018). Low-Intensity exercise with blood flow restriction increases muscle strength without altering hsCRP and Fibrinogen levels in healthy subjects. Chin J Physiol.

[45] Letieri RV, Teixeira AM, Furtado GE, Lamboglia CG, Rees JL, Gomes BB (2018). Effect of 16 weeks of resistance exercise and detraining comparing two methods of blood flow restriction in muscle strength of healthy older women: a randomized controlled trial. Exp Gerontol.

[46] Libardi CA, Chacon-Mikahil MPT, Cavaglieri CR, Tricoli V, Roschel H, Vechin FC (2015). Effect of concurrent training with blood flow restriction in the elderly. Int J Sports Med.

[47] Lixandrao ME, Ugrinowitsch C, Laurentino G, Libardi CA, Aihara AY, Cardoso FN (2015). Effects of exercise intensity and occlusion pressure after 12 weeks of resistance training with blood-flow restriction. Eur J Appl Physiol.

[48] Luebbers PE, Witte EV, Oshel JQ, Butler MS (2019). Effects of practical blood flow restriction training on adolescent lower-body strength. J Strength Cond Res.

[49] May AK, Russell AP, Della Gatta PA, Warmington SA (2022). Muscle adaptations to heavy-load and blood flow restriction resistance training methods. Front Physiol.

[50] Mendonca GV, Vila-Chã C, Teodósio C, Goncalves AD, Freitas SR, Mil-Homens P (2021). Contralateral training effects of low-intensity blood-flow restricted and high-intensity unilateral resistance training. Eur J Appl Physiol.

[51] Morley WN, Ferth S, Debenham MIB, Boston M, Power GA, Burr JF (2021). Training response to 8 weeks of blood flow restricted training is not improved by preferentially altering tissue hypoxia or lactate accumulation when training to repetition failure. Appl Physiol Nutr Metab.

[52] Ramis TR, de Muller CHL, Boeno FP, Teixeira BC, Rech A, Pompermayer MG (2020). Effects of traditional and vascular restricted strength training program with equalized volume on isometric and dynamic strength, muscle thickness, electromyographic activity, and endothelial function adaptations in young adults. J Strength Cond Res.

[53] Sharifi S, Monazzami A, Nikousefat Z, Heyrani A, Yari K (2020). The acute and chronic effects of resistance training with blood flow restriction on hormonal responses in untrained young men: a comparison of frequency. Cell Mol Biol.

[54] Shiromaru FF, de Painelli VS, Silva-Batista C, Longo AR, Lasevicius T, Schoenfeld BJ (2019). Differential muscle hypertrophy and edema responses between high-load and low-load exercise with blood flow restriction. Scand J Med Sci Sports.

[55] Sousa JBC, Neto GR, Santos HH, Araújo JP, Silva HG, Cirilo-Sousa MS (2017). Effects of strength training with blood flow restriction on torque, muscle activation and local muscular endurance in healthy subjects. Biol Sport.

[56] Sugiarto D, Andriati A, Laswati H, Kimura H (2017). Comparison of the increase of both muscle strength and hypertrophy of biceps brachii muscle in strengthening exercise with low-intensity resistance training with and without the application of blood flow restriction and high-intensity resistance training. Bali Med J.

[57] Teixeira EL, de Painelli VS, Schoenfeld BJ, Silva-Batista C, Longo AR, Aihara AY (2022). Perceptual and neuromuscular responses adapt similarly between high-load resistance training and low-load resistance training with blood flow restriction. J Strength Cond Res.

[58] Thiebaud RS, Loenneke JP, Fahs CA, Rossow LM, Kim D, Abe T (2013). The effects of elastic band resistance training combined with blood flow restriction on strength, total bone-free lean body mass and muscle thickness in postmenopausal women. Clin Physiol Funct Imaging.

[59] Shanghua LI, Zhiyun LIU (2020). Biomechanical study on influence of different resistance training combined with blood flow restriction methods on leg muscle volume. J Southwest China Norm Univ Nat Sci Ed.

[60] Li Z, Yu S, Lou H, Wang Y (2022). Effect of KAATSU resistance training on body limb circumference, maximum strength and agility of college student male tennis players. Fujian Sports Sci Technol.

[61] Wang M, Li Z, Wei W, Zhao Z, Chen C, Huang J (2019). Empirical study of KAATSU training effect on lower limb of male elite handball player. China Sport Sci Technol.

[62] Zhang J, Ye J, Wu J (2022). The effect of blood flow restriction with different intensities of strength training on muscle strength and explosive jump performance. Sichuan Sports Sci.

[63] Kataoka R, Vasenina E, Hammert WB, Ibrahim AH, Dankel SJ, Buckner SL (2022). Muscle growth adaptations to high-load training and low-load training with blood flow restriction in calf muscles. Eur J Appl Physiol.

[64] Kim S, Sherk VD, Bemben MG, Bemben DA (2012). Effects of short term low intensity resistance training with blood flow restriction on bone markers and muscle cross-sectional area in young men. Int J Exerc Sci.

[65] Centner C, Jerger S, Lauber B, Seynnes O, Friedrich T, Lolli D (2023). Similar patterns of tendon regional hypertrophy after low-load blood flow restriction and high-load resistance training. Scand J Med Sci Sports.

[66] Araujo Pessôa DE, K, Cholewa JM, Sousasilva R, Xia Z, Zagatto AM, Lancha-Jr AH,  (2023). Does beta-alanine supplementation potentiate muscle performance following 6 weeks of blood flow restriction or traditional resistance training?. Int J Exerc Sci.

[67] Horiuchi M, Stoner L, Poles J (2023). The effect of four weeks blood flow restricted resistance training on macro- and micro-vascular function in healthy, young men. Eur J Appl Physiol.

[68] Judd K, Morales C, White M, Wilkie K, Faller J, Ives SJ (2023). The effects of accessory blood flow restriction training on muscle size and strength in division III soccer athletes: a preliminary ecological study. Int J Exerc Sci.

[69] Reece TM, Godwin JS, Strube MJ, Ciccone AB, Stout KW, Pearson JR (1985). Myofiber hypertrophy adaptations following 6 weeks of low-load resistance training with blood flow restriction in untrained males and females. J Appl Physiol Bethesda Md.

[70] Sousa-Silva R, Cholewa JM, Pessôa KDA, Xia Z, Lauver JD, Rossi FE (2023). Creatine supplementation combined with blood flow restriction training enhances muscle thickness and performance: a randomized, placebo-controlled, and double-blind study. Appl Physiol Nutr Metab.

[71] Wang Z, Atakan MM, Acar B, Xiong R, Peng L (2023). Effects of 4-week low-load resistance training with blood flow restriction on muscle strength and left ventricular function in young swimmers: a pilot randomized trial. J Hum Kinet.

[72] Jukic I, Van Hooren B, Ramos AG, Helms ER, McGuigan MR, Tufano JJ (2021). The effects of set structure manipulation on chronic adaptations to resistance training: a systematic review and meta-analysis. Sports Med Auckl NZ.

[73] Borenstein M, Hedges LV, Higgins JPT, Rothstein HR (2010). A basic introduction to fixed-effect and random-effects models for meta-analysis. Res Synth Methods.

[74] Higgins JPT, Thompson SG, Deeks JJ, Altman DG (2003). Measuring inconsistency in meta-analyses. BMJ.

[75] Schoenfeld BJ, Ogborn D, Krieger JW (2017). Dose-response relationship between weekly resistance training volume and increases in muscle mass: a systematic review and meta-analysis. J Sports Sci.

[76] Takarada Y, Takazawa H, Sato Y, Takebayashi S, Ishii N (2000). Effects of resistance exercise combined with moderate vascular occlusion on muscular function in humans. J Appl Physiol.

[77] Coe R. It’s the effect size, stupid what effect size is and why it is important. In: Annual conference of the British educational research association. England: University of Exeter; 2002.

[78] Ratamess NA (2012). ACSM’s foundations of strength training and conditioning.

[79] Hakkinen K, Komi PV, Alen M, Kauhanen H (1987). EMG, muscle fibre and force production characteristics during a 1 year training period in elite weight-lifters. Eur J Appl Physiol Occup Physiol.

[80] Abe T, Kawamoto K, Yasuda T, Kearns CF, Midorikawa T, Sato Y (2005). Eight days KAATSU-resistance training improved sprint but not jump performance in collegiate male track and field athletes. Int J KAATSU Train Res.

[81] Denadai BS, Oliveira F, Camarda S, Ribeiro L, Greco CC (2017). Effects of low-load resistance training with blood flow restriction on muscle size and strength of professional soccer players with muscle imbalance. Int J Appl Exerc Physiol.

[82] Schoenfeld BJ (2013). Potential mechanisms for a role of metabolic stress in hypertrophic adaptations to resistance training. Sports Med Auckl.

[83] Duchateau J, Stragier S, Baudry S, Carpentier A (2021). Strength training: in search of optimal strategies to maximize neuromuscular performance. Exerc Sport Sci Rev.

[84] Kon M, Ikeda T, Homma T, Suzuki Y (2012). Effects of low-intensity resistance exercise under acute systemic hypoxia on hormonal responses. J Strength Cond Res.

[85] Nishimura A, Sugita M, Kato K, Fukuda A, Sudo A, Uchida A (2010). Hypoxia increases muscle hypertrophy induced by resistance training. Int J SPORTS Physiol Perform.

[86] Christiansen D, Murphy RM, Bangsbo J, Stathis CG, Bishop DJ (2018). Increased FXYD1 and PGC-1α mRNA after blood flow-restricted running is related to fibre type-specific AMPK signalling and oxidative stress in human muscle. Acta Physiol Oxf Engl.

[87] Moore DR, Burgomaster KA, Schofield LM, Gibala MJ, Sale DG, Phillips SM (2004). Neuromuscular adaptations in human muscle following low intensity resistance training with vascular occlusion. Eur J Appl Physiol.

[88] Takarada Y, Nakamura Y, Aruga S, Onda T, Miyazaki S, Ishii N (2000). Rapid increase in plasma growth hormone after low-intensity resistance exercise with vascular occlusion. J Appl Physiol.

[89] Yasuda T, Brechue W, Fujita T, Shirakawa J, Sato Y, Abe T (2009). Muscle activation during low-intensity muscle contractions with restricted blood flow. J Sports Sci.

[90] Lauver JD, Cayot TE, Rotarius T, Scheuermann BW (2017). The effect of eccentric exercise with blood flow restriction on neuromuscular activation, microvascular oxygenation, and the repeated bout effect. Eur J Appl Physiol.

[91] Bordessa JM, Hearn MC, Reinfeldt AE, Smith TA, Baweja HS, Levy SS (2021). Comparison of blood flow restriction devices and their effect on quadriceps muscle activation. Phys Ther Sport.

[92] Cook SB, Murphy BG, Labarbera KE (2013). Neuromuscular function after a bout of low-load blood flow-restricted exercise. Med Sci Sports Exerc.

[93] Manini TM, Clark BC (2009). Blood flow restricted exercise and skeletal muscle health. Exerc Sport Sci Rev.

[94] Loenneke JP, Kim D, Fahs CA, Thiebaud RS, Abe T, Larson RD (2015). Effects of exercise with and without different degrees of blood flow restriction on torque and muscle activation. Muscle Nerve.

[95] Scott B, Slattery K, Sculley D, Dascombe B (2014). Hypoxia and resistance exercise: a comparison of localized and systemic methods. Sports Med.

[96] Rutherford OM, Jones DA (1986). The role of learning and coordination in strength training. Eur J Appl Physiol.

[97] Geng Y, Zhang L, Wu X (2022). Effects of blood flow restriction training on blood perfusion and work ability of muscles in Elite Para-alpine Skiers. Med Sci Sports Exerc.

[98] Scott BR, Loenneke JP, Slattery KM, Dascombe BJ (2016). Blood flow restricted exercise for athletes: a review of available evidence. J Sci Med Sport.

