# Supplementary material for: Potential Moderators of the Effects of Blood Flow Restriction Training on Muscle Strength and Hypertrophy: A Meta-analysis Based on a Comparison with High-Load Resistance Training
Source: Sports Med Open. 2024 May 22;10:58. doi: 10.1186/s40798-024-00719-3 (PMC11109065; doi:10.1186/s40798-024-00719-3)

Electronic Supplementary Material Appendix Tables

Table S1 Literature search strategy

English terms	“Kaatsu training”，“practical Kaatsu training”，“practical blood flow restriction training”, “blood flow restriction training”，“resistance training associated with blood flow restriction”，“strength training associated with blood flow restriction”，“low-load resistance training associated with blood flow restriction”，“low-intensity associated with blood flow restriction”, “blood flow restriction therapy”，“blood flow restriction therapies”，“BFR Therapy”，“BFR Therapies”，“Blood Flow Restriction Training”， and “Blood Flow Restriction Exercise”
Chinese terms	“血流限制”(blood flow restriction), “血流限制训练”(blood flow restriction training) and “加压训练”(Kaatsu training).
Example for Web of Science search strategy	
(TI=(((“blood Flow Restriction” OR Kaatsu) AND (Training OR Exercise)) OR BFR Therap*)) OR AB=(((“blood Flow Restriction” OR Kaatsu) AND (Training OR Exercise)) OR BFR Therap*)	

Table S2 PEDro quality assessment of the included studies.

Study	2	3	4	5	6	7	8	9	10	11	Total
**Karabulut et al.2010 [9]**	1	0	1	1	1	0	1	1	1	1	8
**Yasuda et al.2011 [11]**	1	0	1	0	0	0	1	1	1	1	6
**Clark et al.2011 [14]**	1	0	0	0	0	1	1	1	1	1	6
**Laurentino et al.2012 [15]**	1	0	1	0	0	0	1	1	1	1	6
**Kim et al.2012 [64]**	1	0	1	0	0	0	1	1	1	1	6
**Kim et al.2009 [40]**	1	0	1	0	0	0	1	1	1	1	6
**Martin-Hernandez et al.2013 [12]**	1	0	1	0	0	0	1	1	1	1	6
**Thiebaud et al.2013 [58]**	0	0	1	0	0	0	1	1	1	1	5
**Ozaki et al.2013 [16]**	1	0	1	0	0	0	1	1	1	1	6
**Lixandrao et al.2015 [47]**	1	0	0	0	0	0	1	1	1	1	5
**Ellefsen et al.2015 [17]**	1	0	0	0	0	1	1	1	1	1	6
**Vechin et al.2015 [13]**	1	0	0	0	0	0	1	1	1	1	5
**Cook et al.2017 [34]**	1	0	0	0	0	1	1	1	1	1	6
**Kim et al.2017a [41]**	1	0	1	0	0	0	1	1	1	1	6
**Sousa et al.2017 [55]**	1	0	1	0	0	0	1	1	1	1	6
**Kim et al.2017b [42]**	1	0	1	0	0	0	1	1	1	1	6
**Jessee et al.2018 [39]**	1	0	1	0	0	1	1	1	1	1	7
**Laswati et al.2018 [44]**	1	0	1	0	0	0	1	1	1	1	6
**Letieri et al.2018 [45]**	1	1	1	1	0	0	1	1	1	1	8
**Cook et al.2018 [35]**	1	0	1	0	0	0	1	1	1	1	6
**Luebbers et al.2019 [48]**	1	0	1	0	0	0	1	1	1	1	6
**Shiromaru et al.2019 [54]**	1	0	1	0	0	1	1	1	1	1	7
**Centner et al.2019 [33]**	1	1	1	0	0	1	0	1	1	1	7
**Bjornsen et al.2019 [26]**	1	0	1	0	0	1	1	1	1	1	7
**de Lemos Muller et al.2019 [37]**	1	0	0	0	0	0	1	1	1	1	5
**Ramis et al.2020 [52]**	1	0	1	0	0	1	1	1	1	1	7
**Sharifi et al.2020 [53]**	1	0	0	0	0	0	1	1	1	1	5
**Buckner et al.2020 [32]**	1	0	1	0	0	1	1	1	1	1	7
**Teixeira et al.2020 [57]**	1	0	1	0	0	0	0	1	1	1	5
**Fernandes et al.2020 [38]**	1	0	1	0	0	0	1	1	1	1	6
**Davids et al.2021 [36]**	1	0	1	0	0	1	1	1	1	1	7
**Morley et al.2021 [51]**	1	0	1	0	0	0	0	1	1	1	5
**Mendonca et al.2021 [50]**	1	0	1	0	0	0	1	1	1	1	6
**Kataoka et al.2022 [63]**	1	0	1	0	0	1	1	1	1	1	7
**Korkmaz et al.2022 [43]**	0	0	1	0	0	1	1	1	1	1	6
**Centner et al.2022 [28]**	1	0	1	0	0	1	0	1	1	1	6
**May et al.2022 [49]**	1	0	1	0	0	0	1	1	1	1	6
**Libardi et al.2015 [46]**	1	0	1	0	0	0	0	1	1	1	5
**Li Zhiyuan et al.2019 [18]**	1	0	1	0	0	0	0	1	1	1	5
**Wang Mingbo et al.2019 [61]**	1	0	1	0	0	0	0	1	1	1	5
**Li Zhiyuan et al.2022 [60]**	1	0	1	0	0	0	0	1	1	1	5
**Li Shanghua et al.2020 [59]**	1	0	1	0	0	0	0	1	1	1	5
**Zhang Junjie et al.2022 [62]**	1	0	1	0	0	0	0	1	1	1	5

**Che Tongtong et al.2022 [19]**	1	0	1	0	0	0	0	1	1	1	5
**Sugiarto et al.2017 [56]**	1	0	1	0	0	0	0	1	1	1	5
**Kubo et al.2006 [10]**	1	0	1	0	0	0	0	1	1	1	5
**Centner et al.2023[65]**	1	0	1	0	0	1	1	1	1	1	7
**De Araujo et al.2023[66]**	1	1	1	1	0	1	0	1	1	1	8
**Horiuchi et al.2023[67]**	1	0	1	0	0	0	1	1	1	1	6
**Judd et al.2023[68]**	1	0	1	0	0	0	1	1	1	1	6
**Reece et al.2023[69]**	1	0	1	0	0	0	1	1	1	1	6
**Sousa-Silva et al.2023[70]**	1	1	1	0	0	1	1	1	1	1	8
**Wang et al.2023[71]**	1	0	1	0	0	0	1	1	1	1	6

1 = criterion is satisfied; 0 = criterion not satisfied

2 = subjects were randomly allocated to groups (in a crossover study, subjects were randomly allocated and order in which treatments were received

3 = allocation was concealed

4 = the groups were similar at baseline regarding the most important prognostic indicators

5 = there was blinding of all subjects

6 = there was blinding of all therapists who administered the therapy

7 = there was blinding of all assessors who measured at least one key outcome

8 = measures of at least one key outcome were obtained from more than 85% of the subjects initially allocated to groups

9 = all subjects from whom outcome measures were available received the treatment or control condition as allocated or, where this was not the case, data for at least one key outcome were analyzed by "intention to treat"

10 = the results of between-group statistical comparisons were reported for at least one key outcome

11 = the study provided both point measures and measures of variability for at least one key outcome

Electronic Supplementary Material Appendix Figures

Fig. S1 Forest plot of the effect size difference between BFR-RT versus HL-RT for muscle strength according to gender in the trained individuals. The different capital letters (i.e. A, B) after the reference number are used to represent different training protocols for the same study. Hedges'g represents effect size difference. Red diamonds represent overall Hedges'g of subgroups. *1rm* 1RM test, *BFR-RT* blood-flow restriction low-load resistance training, *CI* confidence interval, *Combined* mean of multiple outcomes from the same training protocol, *HL-RT* high-load resistance training.

Group by GENDER	Study name	Outcome	Hedges' g	Standard error	Lower limit	Upper limit
Female	CHE Tongtong et al.[19]	Combined	0.007	0.480	-0.935	0.948
Female	Judd et al.[68]B	1rm	1.716	0.721	0.304	3.128
Female			0.682	0.572	-0.440	1.804
Male	Judd et al.[68]A	1rm	2.437	0.866	0.740	4.133
Male	Korkmaz et al.[43]	Combined	1.401	0.453	0.513	2.289
Male	LI Shanghua et al.[59]	Combined	0.599	0.415	-0.213	1.412
Male	LI Zhiyuan et al.[18]	Combined	0.865	0.505	-0.125	1.854
Male	LI Zhiyuan et al.[60]	Combined	0.268	0.433	-0.580	1.116
Male	Luebbers et al.[48]	1rm	0.724	0.478	-0.212	1.660
Male	Wang et al.[71]	1rm	0.509	0.481	-0.434	1.452
Male	WANG Mingbo et al.[61]	Combined	0.910	0.485	-0.042	1.861
Male	ZHANG Junjie et al.[62]A	1rm	-0.870	0.497	-1.845	0.104
Male	ZHANG Junjie et al.[62]B	1rm	0.112	0.473	-0.816	1.039
Male			0.609	0.236	0.146	1.072

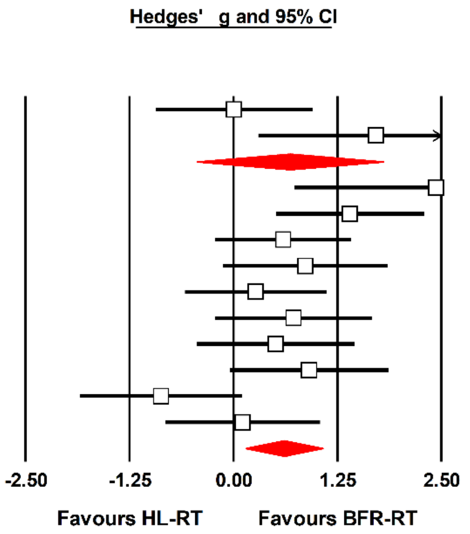

Fig. S2 Forest plot of the effect size difference between BFR-RT versus HL-RT for muscle strength according to limbs in the trained individuals. The different capital letters (i.e. A, B) after the reference number are used to represent different training protocols for the same study. Hedges'g represents effect size difference. Red diamonds represent overall Hedges'g of subgroups. *1rm* 1RM test, *BFR-RT* blood-flow restriction low-load resistance training, *CI* confidence interval, *Combined* mean of multiple outcomes from the same training protocol, *HL-RT* high-load resistance training.

Group by LIMBS	Study name	Outcome	Statistics for each study: Hedges' g	Standard error	Lower limit	Upper limit
Lower limbs	Bjornsen et al.[26]	Combined	0.188	0.470	-0.732	1.109
Lower limbs	CHE Tongtong et al.[19]	Combined	0.007	0.480	-0.935	0.948
Lower limbs	Davids et al.[36]	Combined	-0.322	0.423	-1.150	0.506
Lower limbs	Korkmaz et al.[43]	Combined	1.401	0.453	0.513	2.289
Lower limbs	LI Shanghua et al.[59]	Combined	0.599	0.415	-0.213	1.412
Lower limbs	LI Zhiyuan et al.[18]	Combined	0.865	0.505	-0.125	1.854
Lower limbs	LI Zhiyuan et al.[60]B	1rm	0.545	0.437	-0.311	1.401
Lower limbs	Luebbers et al.[48]	1rm	0.724	0.478	-0.212	1.660
Lower limbs	Wang et al.[71]	1rm	0.509	0.481	-0.434	1.452
Lower limbs	WANG Mingbo et al.[61]	Combined	0.910	0.485	-0.042	1.861
Lower limbs	ZHANG Junjie et al.[62]A	1rm	-0.870	0.497	-1.845	0.104
Lower limbs	ZHANG Junjie et al.[62]B	1rm	0.112	0.473	-0.816	1.039
Lower limbs			0.391	0.198	0.003	0.779
Upper limbs	Judd et al.[68]A	1rm	1.716	0.721	0.304	3.128
Upper limbs	Judd et al.[68]B	1rm	2.437	0.866	0.740	4.133
Upper limbs	LI Zhiyuan et al.[60]A	1rm	-0.009	0.428	-0.848	0.831
Upper limbs			1.011	0.467	0.095	1.926

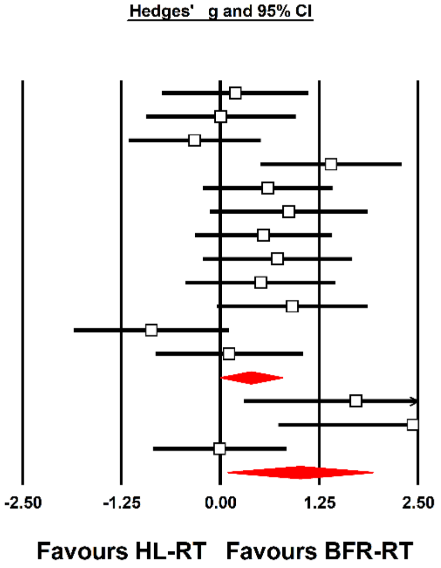

Fig. S3 Forest plot of the effect size difference between BFR-RT versus HL-RT for muscle strength according to training duration in the trained individuals. The different capital letters (i.e. A, B) after the reference number are used to represent different training protocols for the same study. Hedges'g represents effect size difference. Red diamonds represent overall Hedges'g of subgroups. *1rm* 1RM test, *BFR-RT* blood-flow restriction low-load resistance training, *CI* confidence interval, *Combined* mean of multiple outcomes from the same training protocol, *HL-RT* high-load resistance training.

Group by DURATION	Study name	Outcome	Statistics for each study: Hedges' g	Standard error	Lower limit	Upper limit
<=4wk	Bjornsen et al.[26]	Combined	0.188	0.470	-0.732	1.109
<=4wk	Judd et al.[68]B	1rm	1.716	0.721	0.304	3.128
<=4wk	LI Zhiyuan et al.[18]	Combined	0.865	0.505	-0.125	1.854
<=4wk	Wang et al.[71]	1rm	0.509	0.481	-0.434	1.452
<=4wk			0.724	0.371	-0.004	1.452
=5-8wk	CHE Tongtong et al.[19]	Combined	0.007	0.480	-0.935	0.948
=5-8wk	Judd et al.[68]A	1rm	2.437	0.866	0.740	4.133
=5-8wk	Korkmaz et al.[43]	Combined	1.401	0.453	0.513	2.289
=5-8wk	LI Shanghua et al.[59]	Combined	0.599	0.415	-0.213	1.412
=5-8wk	LI Zhiyuan et al.[60]	Combined	0.268	0.433	-0.580	1.116
=5-8wk	Luebbers et al.[48]	1rm	0.724	0.478	-0.212	1.660
=5-8wk	WANG Mingbo et al.[61]	Combined	0.910	0.485	-0.042	1.861
=5-8wk	ZHANG Junjie et al.[62]A	1rm	-0.870	0.497	-1.845	0.104
=5-8wk	ZHANG Junjie et al.[62]B	1rm	0.112	0.473	-0.816	1.039
=5-8wk			0.520	0.238	0.053	0.987

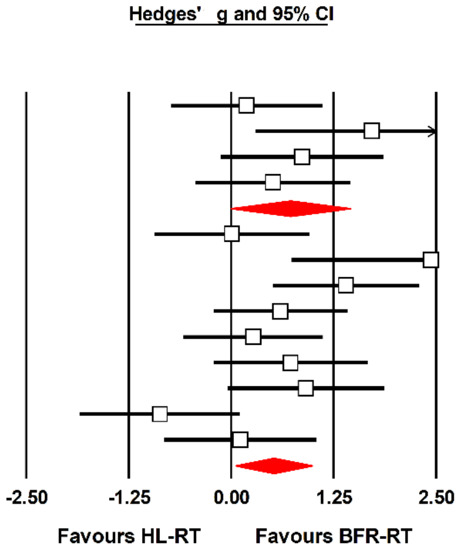

Fig. S4 Forest plot of the effect size difference between BFR-RT versus HL-RT for muscle strength according to training frequency in the trained individuals. The different capital letters (i.e. A, B) after the reference number are used to represent different training protocols for the same study. Hedges'g represents effect size difference. Red diamonds represent overall Hedges'g of subgroups. *1rm* 1RM test, *BFR-RT* blood-flow restriction low-load resistance training, *CI* confidence interval, *Combined* mean of multiple outcomes from the same training protocol, *HL-RT* high-load resistance training.

Group by FREQUENCY	Study name	Outcome	Statistics for each study			
			Hedges' g	Standard error	Lower limit	Upper limit
2/wk	Judd et al.[68]A	1rm	2.437	0.866	0.740	4.133
2/wk	Judd et al.[68]B	1rm	1.716	0.721	0.304	3.128
2/wk	Korkmaz et al.[43]	Combined	1.401	0.453	0.513	2.289
2/wk	LI Zhiyuan et al.[60]	Combined	0.268	0.433	-0.580	1.116
2/wk	Luebbers et al.[48]	1rm	0.724	0.478	-0.212	1.660
2/wk	ZHANG Junjie et al.[62]A	1rm	-0.870	0.497	-1.845	0.104
2/wk	ZHANG Junjie et al.[62]B	1rm	0.112	0.473	-0.816	1.039
2/wk			0.660	0.300	0.072	1.248
3/wk	CHE Tongtong et al.[19]	Combined	0.007	0.480	-0.935	0.948
3/wk	Davids et al.[36]	Combined	-0.322	0.423	-1.150	0.506
3/wk	LI Shanghua et al.[59]	Combined	0.599	0.415	-0.213	1.412
3/wk	LI Zhiyuan et al.[18]	Combined	0.865	0.505	-0.125	1.854
3/wk	Wang et al.[71]	1rm	0.509	0.481	-0.434	1.452
3/wk	WANG Mingbo et al.[61]	Combined	0.910	0.485	-0.042	1.861
3/wk			0.415	0.303	-0.179	1.010

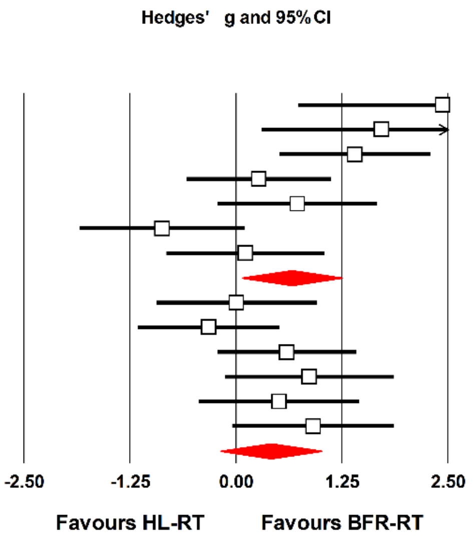

Fig. S5 Forest plot of the effect size difference between BFR-RT versus HL-RT for muscle strength according to test specificity in the trained individuals. The different capital letters (i.e. A, B) after the reference number are used to represent different training protocols for the same study. Hedges'g represents effect size difference. Red diamonds represent overall Hedges'g of subgroups. *1rm* 1RM test, *BFR-RT* blood-flow restriction low-load resistance training, *CI* confidence interval, *Combined* mean of multiple outcomes from the same training protocol, *HL-RT* high-load resistance training, *mvc* isometric or isokinetic tests, *-ns* non-specific test, *-s* specific test.

Group by TEST TYPE	Study name	Outcome	Statistics for each study: Hedges' g	Standard error	Lower limit	Upper limit
Non-specific test	Bjornsen et al.[26]-ns	mvc	0.665	0.475	-0.266	1.597
Non-specific test	CHE Tongtong et al.[19]-ns	Combined	-0.079	0.479	-1.019	0.860
Non-specific test	Davids et al.[36]-ns	mvc	-0.403	0.424	-1.234	0.428
Non-specific test	Korkmaz et al.[43]	Combined	1.401	0.453	0.513	2.289
Non-specific test	LI Shanghua et al.[59]	Combined	0.812	0.466	-0.101	1.725
Non-specific test	WANG Mingbo et al.[61]	Combined	0.910	0.485	-0.042	1.861
Non-specific test			0.541	0.282	-0.011	1.094
Specific test	Bjornsen et al.[26]-s	1rm	-0.289	0.464	-1.198	0.621
Specific test	CHE Tongtong et al.[19]-s	1rm	0.695	0.488	-0.262	1.652
Specific test	Davids et al.[36]-s	1rm	-0.241	0.421	-1.066	0.585
Specific test	Judd et al.[68]A	1rm	2.437	0.866	0.740	4.133
Specific test	Judd et al.[68]B	1rm	1.716	0.721	0.304	3.128
Specific test	LI Zhiyuan et al.[18]	1rm	0.224	0.474	-0.706	1.154
Specific test	LI Zhiyuan et al.[60]	Combined	0.268	0.433	-0.580	1.116
Specific test	Luebbers et al.[48]	1rm	0.724	0.478	-0.212	1.660
Specific test	Wang et al.[71]	1rm	0.509	0.481	-0.434	1.452
Specific test	ZHANG Junjie et al.[62]A	1rm	-0.870	0.497	-1.845	0.104
Specific test	ZHANG Junjie et al.[62]B	1rm	0.112	0.473	-0.816	1.039
Specific test			0.330	0.218	-0.098	0.758

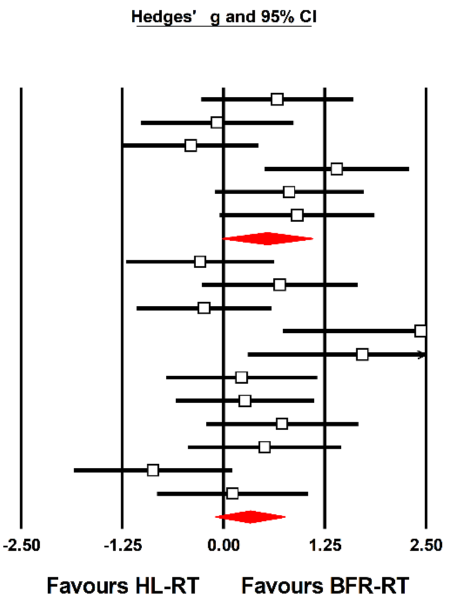

Fig. S6 Forest plot of the effect size difference between BFR-RT versus HL-RT for muscle strength according to gender in the untrained individuals. The different capital letters (i.e. A, B) after the reference number are used to represent different training protocols for the same study. Hedges'g represents effect size difference. Red diamonds represent overall Hedges'g of subgroups. *1rm* 1RM test, *BFR-RT* blood-flow restriction low-load resistance training, *CI* confidence interval, *Combined* mean of multiple outcomes from the same training protocol, *HL-RT* high-load resistance training, *mvc* isometric or isokinetic tests.

Group by GENDER	Study name	Outcome	Statistics for each study: Hedges' g	Standard error	Lower limit	Upper limit
Female	Fernandes et al.[38]	Combined	-0.258	0.369	-0.980	0.465
Female	Letieri et al.[45]A	Combined	-0.226	0.415	-1.038	0.587
Female	Letieri et al.[45]B	Combined	-1.485	0.478	-2.422	-0.548
Female	Reece et al.[69]B	1rm	-0.899	0.499	-1.876	0.079
Female	Thiebaud et al.[58]	Combined	-0.469	0.523	-1.494	0.556
Female			-0.638	0.297	-1.220	-0.055
Male	Centner et al.[28]	Combined	0.945	0.421	0.119	1.771
Male	Centner et al.[33]	mvc	-0.189	0.391	-0.955	0.576
Male	Centner et al.[65]	1rm	0.208	0.362	-0.502	0.918
Male	De Araujo et al.[66]	1rm	-1.895	0.523	-2.920	-0.871
Male	de Lemos Muller et al.[37]	Combined	-1.894	0.479	-2.834	-0.955
Male	Ellefsen et al.[17]	1rm	-0.327	0.397	-1.106	0.451
Male	Horiuchi et al.[67]	Combined	-0.465	0.401	-1.250	0.320
Male	Karabulut et al.[9]	Combined	-0.263	0.396	-1.040	0.514
Male	Kim et al.[40]	Combined	-0.618	0.442	-1.485	0.248
Male	Kim et al.[41]	Combined	-0.366	0.454	-1.256	0.524
Male	Kubo et al.[10]	mvc	-0.601	0.460	-1.503	0.300
Male	Laswati et al.[44]	mvc	0.735	0.554	-0.350	1.820
Male	Laurentino et al.[15]	1rm	0.840	0.460	-0.061	1.741
Male	Lixandrao et al.[47]A	1rm	-1.087	0.464	-1.995	-0.178
Male	Lixandrao et al.[47]B	1rm	-0.982	0.437	-1.837	-0.126
Male	Lixandrao et al.[47]C	1rm	-0.871	0.485	-1.821	0.079
Male	Lixandrao et al.[47]D	1rm	-0.852	0.460	-1.753	0.050
Male	Martin-Hernandez et al.[12]A	Combined	-0.978	0.450	-1.859	-0.096
Male	Martin-Hernandez et al.[12]B	Combined	-0.705	0.442	-1.571	0.161
Male	May et al.[49]	Combined	-0.186	0.463	-1.093	0.721
Male	Ozaki et al.[16]	1rm	-0.868	0.461	-1.772	0.035
Male	Ramis et al.[52]	Combined	-1.071	0.400	-1.856	-0.287
Male	Reece et al.[69]A	1rm	-1.883	0.614	-3.087	-0.680
Male	Sharifi et al.[53]A	Combined	-0.285	0.475	-1.216	0.647
Male	Sharifi et al.[53]B	Combined	-0.086	0.541	-1.147	0.974
Male	Shiromaru et al.[54]	1rm	-0.524	0.362	-1.233	0.185
Male	Sousa-Silva et al.[70]	1rm	-0.734	0.465	-1.646	0.178
Male	Sugiarto et al.[56]	Combined	0.877	0.563	-0.228	1.981
Male	Teixeira et al.[57]	1rm	-0.501	0.481	-1.444	0.441
Male	Vechin et al.[13]	1rm	-0.692	0.488	-1.649	0.265
Male	Yasuda et al.[11]	Combined	-1.034	0.459	-1.932	-0.135
Male			-0.516	0.120	-0.751	-0.282

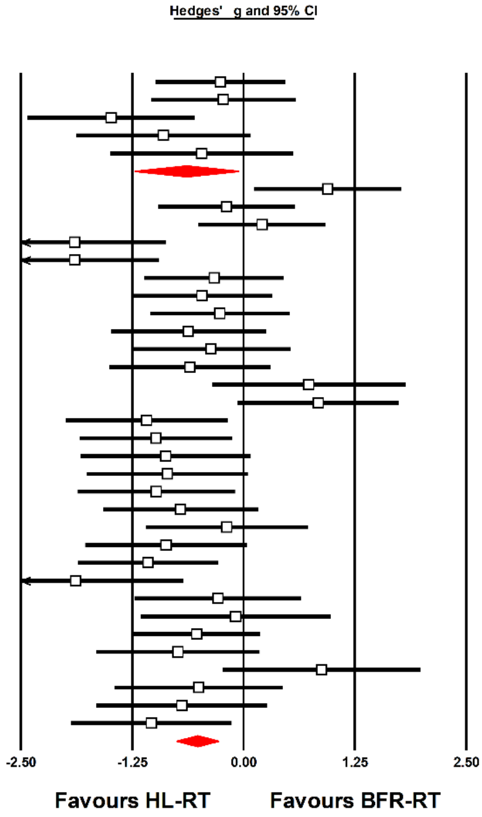

Fig. S7 Forest plot of the effect size difference between BFR-RT versus HL-RT for muscle strength according to age in the untrained individuals. The different capital letters (i.e. A, B, C, D) after the reference number are used to represent different training protocols for the same study. Hedges'g represents effect size difference. Red diamonds represent overall Hedges'g of subgroups. *1rm* 1RM test, *BFR-RT* blood-flow restriction low-load resistance training, *CI* confidence interval, *Combined* mean of multiple outcomes from the same training protocol, *HL-RT* high-load resistance training, *mvc* isometric or isokinetic tests.

Group by AGE	Study name	Outcome	Hedges' g	Standard error	Lower limit	Upper limit
			Statistics for each study			
Old	Cook et al.[34]	Combined	-0.629	0.407	-1.426	0.168
Old	Karabulut et al.[9]	Combined	-0.263	0.396	-1.040	0.514
Old	Kim et al.[42]	mvc	-1.324	0.489	-2.282	-0.366
Old	Letieri et al.[45]A	Combined	-0.226	0.415	-1.038	0.587
Old	Letieri et al.[45]B	Combined	-1.485	0.478	-2.422	-0.548
Old	Libardi et al.[46]	1rm	-0.832	0.473	-1.758	0.094
Old	Thiebaud et al.[58]	Combined	-0.469	0.523	-1.494	0.556
Old	Vechin et al.[13]	1rm	-0.692	0.488	-1.649	0.265
Old			-0.714	0.205	-1.115	-0.312
Young	Buckner et al.[32]A	Combined	-0.534	0.319	-1.159	0.090
Young	Buckner et al.[32]B	Combined	-0.377	0.317	-0.997	0.243
Young	Centner et al.[28]	Combined	0.945	0.421	0.119	1.771
Young	Centner et al.[33]	mvc	-0.189	0.391	-0.955	0.576
Young	Centner et al.[65]	1rm	0.208	0.362	-0.502	0.918
Young	Clark et al.[14]	mvc	-0.487	0.484	-1.436	0.462
Young	Cook et al.[35]	Combined	-0.806	0.558	-1.900	0.287
Young	De Araujo et al.[66]	1rm	-1.895	0.523	-2.920	-0.871
Young	de Lemos Muller et al.[37]	Combined	-1.894	0.479	-2.834	-0.955
Young	Ellefsen et al.[17]	1rm	-0.327	0.397	-1.106	0.451
Young	Fernandes et al.[38]	Combined	-0.258	0.369	-0.980	0.465
Young	Horiuchi et al.[67]	Combined	-0.465	0.401	-1.250	0.320
Young	Jessee et al.[39]A	Combined	-0.709	0.453	-1.598	0.180
Young	Jessee et al.[39]B	Combined	-0.668	0.447	-1.543	0.208
Young	Kim et al.[40]	Combined	-0.618	0.442	-1.485	0.248
Young	Kim et al.[41]	Combined	-0.366	0.454	-1.256	0.524
Young	Kubo et al.[10]	mvc	-0.601	0.460	-1.503	0.300
Young	Laswati et al.[44]	mvc	0.735	0.554	-0.350	1.820
Young	Laurentino et al.[15]	1rm	0.840	0.460	-0.061	1.741
Young	Lixandrao et al.[47]A	1rm	-1.087	0.464	-1.995	-0.178
Young	Lixandrao et al.[47]B	1rm	-0.982	0.437	-1.837	-0.126
Young	Lixandrao et al.[47]C	1rm	-0.871	0.485	-1.821	0.079
Young	Lixandrao et al.[47]D	1rm	-0.852	0.460	-1.753	0.050
Young	Martin-Hernandez et al.[12]A	Combined	-0.978	0.450	-1.859	-0.096
Young	Martin-Hernandez et al.[12]B	Combined	-0.705	0.442	-1.571	0.161
Young	May et al.[49]	Combined	-0.186	0.463	-1.093	0.721
Young	Mendonca et al.[50]	mvc	-0.023	0.355	-0.719	0.674
Young	Morley et al.[51]A	Combined	-0.377	0.521	-1.398	0.644
Young	Morley et al.[51]B	Combined	-0.097	0.522	-1.119	0.925
Young	Ozaki et al.[16]	1rm	-0.868	0.461	-1.772	0.035
Young	Ramis et al.[52]	Combined	-1.071	0.400	-1.856	-0.287
Young	Reece et al.[69]A	1rm	-1.883	0.614	-3.087	-0.680
Young	Reece et al.[69]B	1rm	-0.899	0.499	-1.876	0.079
Young	Sharifi et al.[53]A	Combined	-0.285	0.475	-1.216	0.647
Young	Sharifi et al.[53]B	Combined	-0.086	0.541	-1.147	0.974
Young	Shiromaru et al.[54]	1rm	-0.524	0.362	-1.233	0.185
Young	Sousa et al.[55]	mvc	-1.355	0.469	-2.274	-0.437
Young	Sousa-Silva et al.[70]	1rm	-0.734	0.465	-1.646	0.178
Young	Teixeira et al.[57]	1rm	-0.501	0.481	-1.444	0.441
Young	Yasuda et al.[11]	Combined	-1.034	0.459	-1.932	-0.135
Young			-0.544	0.091	-0.721	-0.367

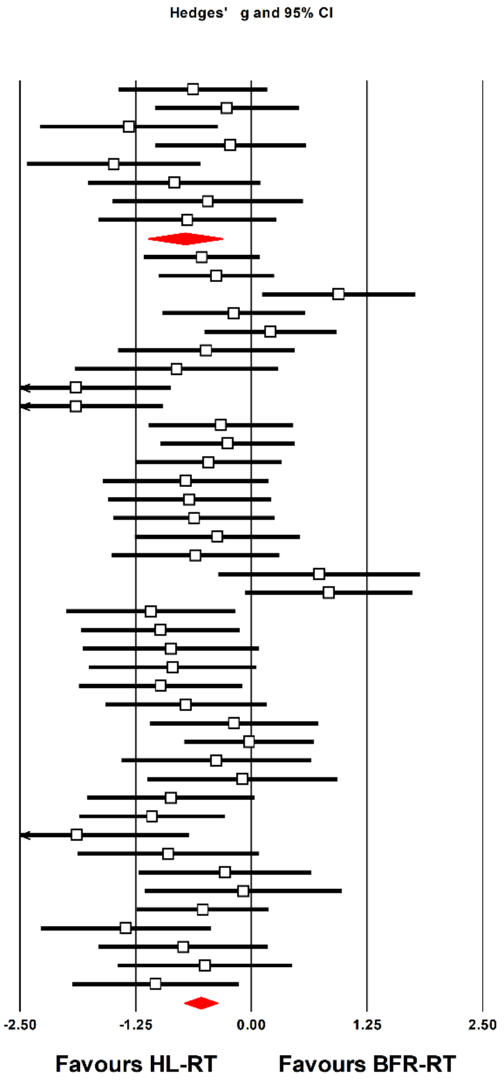

Fig. S8 Forest plot of the effect size difference between BFR-RT versus HL-RT for muscle strength according to limbs in the untrained individuals. The different capital letters (i.e. A, B, C, D) after the reference number are used to represent different training protocols for the same study. Hedges'g represents effect size difference. Red diamonds represent overall Hedges'g of subgroups. *1rm* 1RM test, *BFR-RT* blood-flow restriction low-load resistance training, *CI* confidence interval, *Combined* mean of multiple outcomes from the same training protocol, *HL-RT* high-load resistance training, *mvc* isometric or isokinetic tests, *-l* lower limbs, *-u* upper limbs.

Group by LIMBS	Study name	Outcome	Hedges' g	Standard error	Lower limit	Upper limit
Lower limbs	Centner et al.[28]	Combined	0.945	0.421	0.119	1.771
Lower limbs	Centner et al.[33]	mvc	-0.189	0.391	-0.955	0.576
Lower limbs	Centner et al.[65]	1rm	0.208	0.362	-0.502	0.918
Lower limbs	Clark et al.[14]	mvc	-0.487	0.484	-1.436	0.462
Lower limbs	Cook et al.[34]	Combined	-0.629	0.407	-1.426	0.168
Lower limbs	Cook et al.[35]	Combined	-0.806	0.558	-1.900	0.287
Lower limbs	de Lemos Muller et al.[37]-l	1rm	-2.823	0.546	-3.893	-1.754
Lower limbs	Ellefsen et al.[17]	1rm	-0.327	0.397	-1.106	0.451
Lower limbs	Horiuchi et al.[67]	Combined	-0.465	0.401	-1.250	0.320
Lower limbs	Jessee et al.[39]A	Combined	-0.709	0.453	-1.598	0.180
Lower limbs	Jessee et al.[39]B	Combined	-0.668	0.447	-1.543	0.208
Lower limbs	Karabulut et al.[9]	Combined	-0.263	0.396	-1.040	0.514
Lower limbs	Kim et al.[40]	Combined	-0.618	0.442	-1.485	0.248
Lower limbs	Kubo et al.[10]	mvc	-0.601	0.460	-1.503	0.300
Lower limbs	Laurentino et al.[15]	1rm	0.840	0.460	-0.061	1.741
Lower limbs	Letieri et al.[45]A	Combined	-0.226	0.415	-1.038	0.587
Lower limbs	Letieri et al.[45]B	Combined	-1.485	0.478	-2.422	-0.548
Lower limbs	Libardi et al.[46]	1rm	-0.832	0.473	-1.758	0.094
Lower limbs	Lixandrao et al.[47]A	1rm	-1.087	0.464	-1.995	-0.178
Lower limbs	Lixandrao et al.[47]B	1rm	-0.982	0.437	-1.837	-0.126
Lower limbs	Lixandrao et al.[47]C	1rm	-0.871	0.485	-1.821	0.079
Lower limbs	Lixandrao et al.[47]D	1rm	-0.852	0.460	-1.753	0.050
Lower limbs	Martin-Hernandez et al.[12]A	Combined	-0.978	0.450	-1.859	-0.096
Lower limbs	Martin-Hernandez et al.[12]B	Combined	-0.705	0.442	-1.571	0.161
Lower limbs	May et al.[49]	Combined	-0.186	0.463	-1.093	0.721
Lower limbs	Mendonca et al.[50]	mvc	-0.023	0.355	-0.719	0.674
Lower limbs	Morley et al.[51]A	Combined	-0.377	0.521	-1.398	0.644
Lower limbs	Morley et al.[51]B	Combined	-0.097	0.522	-1.119	0.925
Lower limbs	Ramis et al.[52]-l	Combined	-1.340	0.409	-2.143	-0.538
Lower limbs	Reece et al.[69]A	1rm	-1.883	0.614	-3.087	-0.680
Lower limbs	Reece et al.[69]B	1rm	-0.899	0.499	-1.876	0.079
Lower limbs	Sharifi et al.[53]A-l	1rm	-0.313	0.476	-1.246	0.620
Lower limbs	Sharifi et al.[53]B-l	1rm	-1.573	0.548	-2.647	-0.498
Lower limbs	Shiromaru et al.[54]	1rm	-0.524	0.362	-1.233	0.185
Lower limbs	Sousa et al.[55]	mvc	-1.355	0.469	-2.274	-0.437
Lower limbs	Teixeira et al.[57]	1rm	-0.501	0.481	-1.444	0.441
Lower limbs			-0.618	0.111	-0.836	-0.400
Upper limbs	Buckner et al.[32]A	Combined	-0.534	0.319	-1.159	0.090
Upper limbs	Buckner et al.[32]B	Combined	-0.377	0.317	-0.997	0.243
Upper limbs	De Araujo et al.[66]	1rm	-1.895	0.523	-2.920	-0.871
Upper limbs	de Lemos Muller et al.[37]-u	1rm	-0.965	0.403	-1.755	-0.176
Upper limbs	Fernandes et al.[38]	Combined	-0.258	0.369	-0.980	0.465
Upper limbs	Kim et al.[41]	Combined	-0.366	0.454	-1.256	0.524
Upper limbs	Kim et al.[42]	mvc	-1.324	0.489	-2.282	-0.366
Upper limbs	Laswati et al.[44]	mvc	0.735	0.554	-0.350	1.820
Upper limbs	Ozaki et al.[16]	1rm	-0.868	0.461	-1.772	0.035
Upper limbs	Ramis et al.[52]-u	Combined	-0.802	0.391	-1.569	-0.036
Upper limbs	Sharifi et al.[53]A-u	1rm	-0.256	0.475	-1.187	0.675
Upper limbs	Sharifi et al.[53]B-u	1rm	1.400	0.534	0.354	2.446
Upper limbs	Sousa-Silva et al.[70]	1rm	-0.734	0.465	-1.646	0.178
Upper limbs	Sugiarto et al.[56]	Combined	0.877	0.563	-0.228	1.981
Upper limbs	Thiebaud et al.[58]	Combined	-0.469	0.523	-1.494	0.556
Upper limbs	Vechin et al.[13]	1rm	-0.692	0.488	-1.649	0.265
Upper limbs	Yasuda et al.[11]	Combined	-1.034	0.459	-1.932	-0.135
Upper limbs			-0.477	0.162	-0.794	-0.160

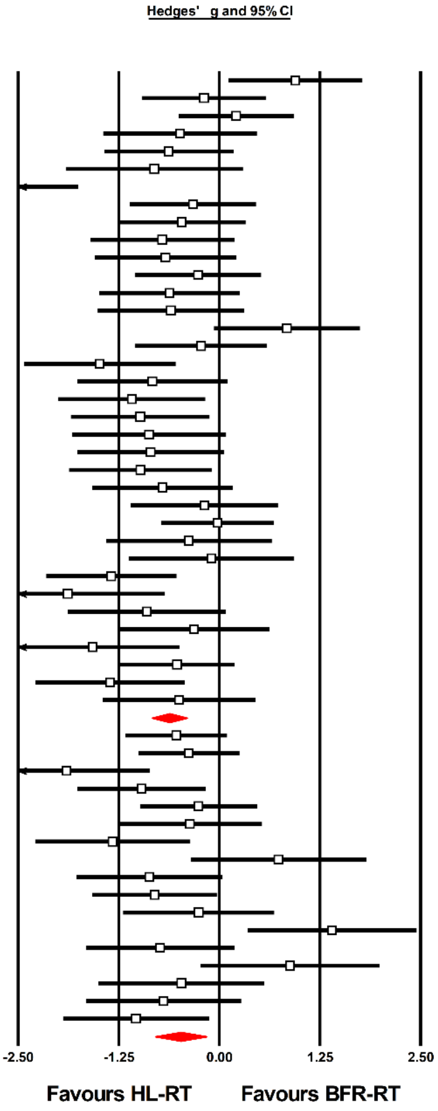

Fig. S9 Forest plot of the effect size difference between BFR-RT versus HL-RT for muscle strength according to training duration in the untrained individuals. The different capital letters (i.e. A, B, C, D) after the reference number are used to represent different training protocols for the same study. Hedges'g represents effect size difference. Red diamonds represent overall Hedges'g of subgroups. 1rm 1RM test, BFR-RT blood-flow restriction low-load resistance training, CI confidence interval, Combined mean of multiple outcomes from the same training protocol, HL-RT high-load resistance training, mvc isometric or isokinetic tests.

Group by DURATION	Study name	Outcome	Statistics for each study: Hedges' g	Standard error	Lower limit	Upper limit
<=4wk	Clark et al.[14]	mvc	-0.487	0.484	-1.436	0.462
<=4wk	Fernandes et al.[38]	Combined	-0.258	0.369	-0.980	0.465
<=4wk	Horiuchi et al.[67]	Combined	-0.465	0.401	-1.250	0.320
<=4wk	Kim et al.[40]	Combined	-0.618	0.442	-1.485	0.248
<=4wk	Kim et al.[42]	mvc	-1.324	0.489	-2.282	-0.366
<=4wk	Mendonca et al.[50]	mvc	-0.023	0.355	-0.719	0.674
<=4wk	Shiromaru et al.[54]	1rm	-0.524	0.362	-1.233	0.185
<=4wk			-0.493	0.216	-0.916	-0.070
=5-8wk	Buckner et al.[32]A	Combined	-0.534	0.319	-1.159	0.090
=5-8wk	Buckner et al.[32]B	Combined	-0.377	0.317	-0.997	0.243
=5-8wk	Cook et al.[35]	Combined	-0.806	0.558	-1.900	0.287
=5-8wk	De Araujo et al.[66]	1rm	-1.895	0.523	-2.920	-0.871
=5-8wk	de Lemos Muller et al.[37]	Combined	-1.894	0.479	-2.834	-0.955
=5-8wk	Jessee et al.[39]A	Combined	-0.709	0.453	-1.598	0.180
=5-8wk	Jessee et al.[39]B	Combined	-0.668	0.447	-1.543	0.208
=5-8wk	Karabulut et al.[9]	Combined	-0.263	0.396	-1.040	0.514
=5-8wk	Kim et al.[41]	Combined	-0.366	0.454	-1.256	0.524
=5-8wk	Laswati et al.[44]	mvc	0.735	0.554	-0.350	1.820
=5-8wk	Laurentino et al.[15]	1rm	0.840	0.460	-0.061	1.741
=5-8wk	Martin-Hernandez et al.[12]A	Combined	-0.978	0.450	-1.859	-0.096
=5-8wk	Martin-Hernandez et al.[12]B	Combined	-0.705	0.442	-1.571	0.161
=5-8wk	May et al.[49]	Combined	-0.186	0.463	-1.093	0.721
=5-8wk	Morley et al.[51]A	Combined	-0.377	0.521	-1.398	0.644
=5-8wk	Morley et al.[51]B	Combined	-0.097	0.522	-1.119	0.925
=5-8wk	Ozaki et al.[16]	1rm	-0.868	0.461	-1.772	0.035
=5-8wk	Ramis et al.[52]	Combined	-1.071	0.400	-1.856	-0.287
=5-8wk	Reece et al.[69]A	1rm	-1.883	0.614	-3.087	-0.680
=5-8wk	Reece et al.[69]B	1rm	-0.899	0.499	-1.876	0.079
=5-8wk	Sharifi et al.[53]A	Combined	-0.285	0.475	-1.216	0.647
=5-8wk	Sharifi et al.[53]B	Combined	-0.086	0.541	-1.147	0.974
=5-8wk	Sousa et al.[55]	mvc	-1.355	0.469	-2.274	-0.437
=5-8wk	Sousa-Silva et al.[70]	1rm	-0.734	0.465	-1.646	0.178
=5-8wk	Sugiarto et al.[56]	Combined	0.877	0.563	-0.228	1.981
=5-8wk	Teixeira et al.[57]	1rm	-0.501	0.481	-1.444	0.441
=5-8wk	Thiebaud et al.[58]	Combined	-0.469	0.523	-1.494	0.556
=5-8wk	Yasuda et al.[11]	Combined	-1.034	0.459	-1.932	-0.135
=5-8wk			-0.593	0.116	-0.820	-0.365
>=9 wk	Centner et al.[28]	Combined	0.945	0.421	0.119	1.771
>=9 wk	Centner et al.[33]	mvc	-0.189	0.391	-0.955	0.576
>=9 wk	Centner et al.[65]	1rm	0.208	0.362	-0.502	0.918
>=9 wk	Cook et al.[34]	Combined	-0.629	0.407	-1.426	0.168
>=9 wk	Ellefsen et al.[17]	1rm	-0.327	0.397	-1.106	0.451
>=9 wk	Kubo et al.[10]	mvc	-0.601	0.460	-1.503	0.300
>=9 wk	Letieri et al.[45]A	Combined	-0.226	0.415	-1.038	0.587
>=9 wk	Letieri et al.[45]B	Combined	-1.485	0.478	-2.422	-0.548
>=9 wk	Libardi et al.[46]	1rm	-0.832	0.473	-1.758	0.094
>=9 wk	Lixandrao et al.[47]A	1rm	-1.087	0.464	-1.995	-0.178
>=9 wk	Lixandrao et al.[47]B	1rm	-0.982	0.437	-1.837	-0.126
>=9 wk	Lixandrao et al.[47]C	1rm	-0.871	0.485	-1.821	0.079
>=9 wk	Lixandrao et al.[47]D	1rm	-0.852	0.460	-1.753	0.050
>=9 wk	Vechin et al.[13]	1rm	-0.692	0.488	-1.649	0.265
>=9 wk			-0.507	0.158	-0.816	-0.198

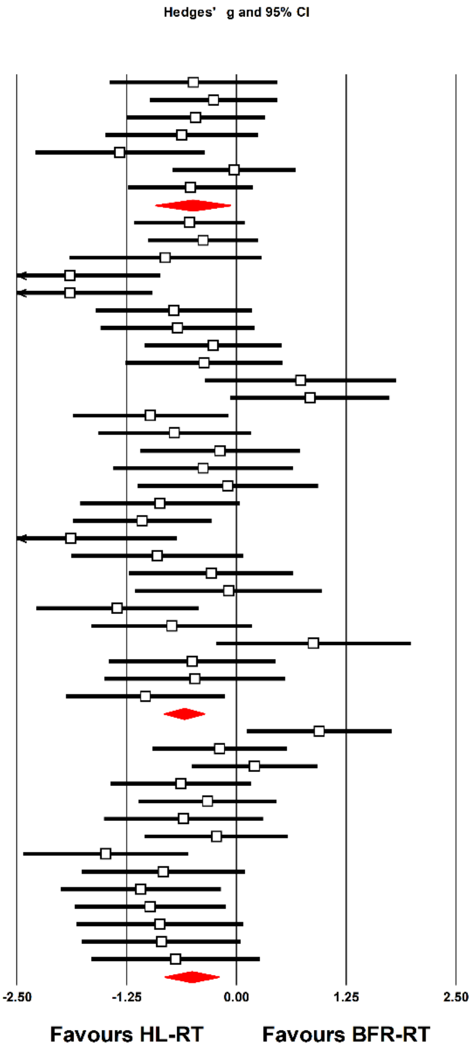

Fig. S10 Forest plot of the effect size difference between BFR-RT versus HL-RT for muscle strength according to training frequency in the untrained individuals. The different capital letters (i.e. A, B, C, D) after the reference number are used to represent different training protocols for the same study. Hedges'g represents effect size difference. Red diamonds represent overall Hedges'g of subgroups. *1rm* 1RM test, *BFR-RT* blood-flow restriction low-load resistance training, *CI* confidence interval, *Combined* mean of multiple outcomes from the same training protocol, *HL-RT* high-load resistance training, *mvc* isometric or isokinetic tests.

Group by FREQUENCY	Study name	Outcome	Statistics for each study			
			Hedges' g	Standard error	Lower limit	Upper limit
2/wk	Buckner et al.[32]A	Combined	-0.534	0.319	-1.159	0.090
2/wk	Buckner et al.[32]B	Combined	-0.377	0.317	-0.997	0.243
2/wk	Cook et al.[34]	Combined	-0.629	0.407	-1.426	0.168
2/wk	De Araujo et al.[66]	1rm	-1.895	0.523	-2.920	-0.871
2/wk	Ellefsen et al.[17]	1rm	-0.327	0.397	-1.106	0.451
2/wk	Jessee et al.[39]A	Combined	-0.709	0.453	-1.598	0.180
2/wk	Jessee et al.[39]B	Combined	-0.668	0.447	-1.543	0.208
2/wk	Laswati et al.[44]	mvc	0.735	0.554	-0.350	1.820
2/wk	Laurentino et al.[15]	1rm	0.840	0.460	-0.061	1.741
2/wk	Libardi et al.[46]	1rm	-0.832	0.473	-1.758	0.094
2/wk	Lixandrao et al.[47]A	1rm	-1.087	0.464	-1.995	-0.178
2/wk	Lixandrao et al.[47]B	1rm	-0.982	0.437	-1.837	-0.126
2/wk	Lixandrao et al.[47]C	1rm	-0.871	0.485	-1.821	0.079
2/wk	Lixandrao et al.[47]D	1rm	-0.852	0.460	-1.753	0.050
2/wk	Martin-Hernandez et al.[12]A	Combined	-0.978	0.450	-1.859	-0.096
2/wk	Martin-Hernandez et al.[12]B	Combined	-0.705	0.442	-1.571	0.161
2/wk	Sousa et al.[55]	mvc	-1.355	0.469	-2.274	-0.437
2/wk	Sousa-Silva et al.[70]	1rm	-0.734	0.465	-1.646	0.178
2/wk	Sugiarto et al.[56]	Combined	0.877	0.563	-0.228	1.981
2/wk	Teixeira et al.[57]	1rm	-0.501	0.481	-1.444	0.441
2/wk	Vechin et al.[13]	1rm	-0.692	0.488	-1.649	0.265
2/wk			-0.596	0.131	-0.853	-0.339
3/wk	Centner et al.[28]	Combined	0.945	0.421	0.119	1.771
3/wk	Centner et al.[33]	mvc	-0.189	0.391	-0.955	0.576
3/wk	Centner et al.[65]	1rm	0.208	0.362	-0.502	0.918
3/wk	Clark et al.[14]	mvc	-0.487	0.484	-1.436	0.462
3/wk	Cook et al.[35]	Combined	-0.806	0.558	-1.900	0.287
3/wk	de Lemos Muller et al.[37]	Combined	-1.894	0.479	-2.834	-0.955
3/wk	Fernandes et al.[38]	Combined	-0.258	0.369	-0.980	0.465
3/wk	Karabulut et al.[9]	Combined	-0.263	0.396	-1.040	0.514
3/wk	Kim et al.[40]	Combined	-0.618	0.442	-1.485	0.248
3/wk	Kim et al.[41]	Combined	-0.366	0.454	-1.256	0.524
3/wk	Kim et al.[42]	mvc	-1.324	0.489	-2.282	-0.366
3/wk	Kubo et al.[10]	mvc	-0.601	0.460	-1.503	0.300
3/wk	Letieri et al.[45]A	Combined	-0.226	0.415	-1.038	0.587
3/wk	Letieri et al.[45]B	Combined	-1.485	0.478	-2.422	-0.548
3/wk	May et al.[49]	Combined	-0.186	0.463	-1.093	0.721
3/wk	Morley et al.[51]A	Combined	-0.377	0.521	-1.398	0.644
3/wk	Morley et al.[51]B	Combined	-0.097	0.522	-1.119	0.925
3/wk	Ozaki et al.[16]	1rm	-0.868	0.461	-1.772	0.035
3/wk	Ramis et al.[52]	Combined	-1.071	0.400	-1.856	-0.287
3/wk	Reece et al.[69]A	1rm	-1.883	0.614	-3.087	-0.680
3/wk	Reece et al.[69]B	1rm	-0.899	0.499	-1.876	0.079
3/wk	Sharifi et al.[53]A	Combined	-0.285	0.475	-1.216	0.647
3/wk	Shiromaru et al.[54]	1rm	-0.524	0.362	-1.233	0.185
3/wk	Thiebaud et al.[58]	Combined	-0.469	0.523	-1.494	0.556
3/wk	Yasuda et al.[11]	Combined	-1.034	0.459	-1.932	-0.135
3/wk			-0.560	0.121	-0.797	-0.323
4/wk	Horiuchi et al.[67]	Combined	-0.465	0.401	-1.250	0.320
4/wk	Mendonca et al.[50]	mvc	-0.023	0.355	-0.719	0.674
4/wk			-0.231	0.388	-0.991	0.528

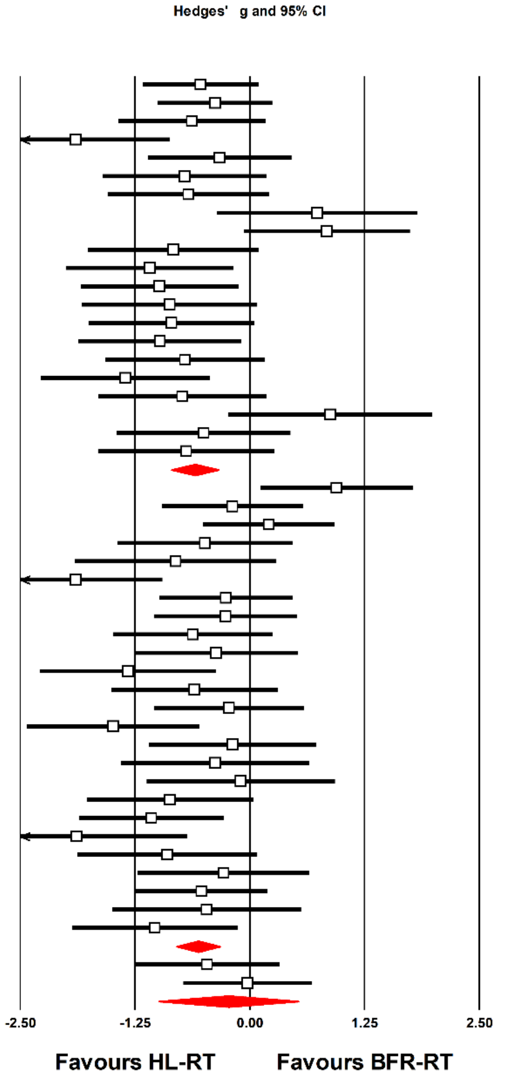

Fig. S11 Forest plot of the effect size difference between BFR-RT versus HL-RT for muscle strength according to test specificity in the untrained individuals. The different capital letters (i.e. A, B, C, D) after the reference number are used to represent different training protocols for the same study. Hedges'g represents effect size difference. Red diamonds represent overall Hedges'g of subgroups. *1rm* 1RM test, *BFR-RT* blood-flow restriction low-load resistance training, *CI* confidence interval, *Combined* mean of multiple outcomes from the same training protocol, *HL-RT* high-load resistance training, *mvc* isometric or isokinetic tests, *-ns* non-specific test, *-s* specific test.

Group by TEST TYPE	Study name	Outcome	Hedges' g	Standard error	Lower limit	Upper limit
Non-specific test	Buckner et al.[32]A-ns	Combined	-0.332	0.313	-0.945	0.281
Non-specific test	Buckner et al.[32]B-ns	Combined	-0.184	0.313	-0.797	0.429
Non-specific test	Centner et al.[33]	mvc	-0.189	0.391	-0.955	0.576
Non-specific test	Clark et al.[14]	mvc	-0.487	0.484	-1.436	0.462
Non-specific test	Cook et al.[34]-ns	mvc	-0.397	0.398	-1.178	0.383
Non-specific test	Cook et al.[35]-ns	mvc	-0.878	0.562	-1.980	0.224
Non-specific test	Fernandes et al.[38]	Combined	-0.258	0.369	-0.980	0.465
Non-specific test	Jessee et al.[39]A-ns	Combined	-0.407	0.437	-1.264	0.451
Non-specific test	Jessee et al.[39]B-ns	Combined	-0.456	0.437	-1.312	0.400
Non-specific test	Kim et al.[41]-ns	Combined	-0.387	0.455	-1.279	0.505
Non-specific test	Kim et al.[42]	mvc	-1.324	0.489	-2.282	-0.366
Non-specific test	Kubo et al.[10]	mvc	-0.601	0.460	-1.503	0.300
Non-specific test	Laswati et al.[44]	mvc	0.735	0.554	-0.350	1.820
Non-specific test	Letieri et al.[45]A	Combined	-0.226	0.415	-1.038	0.587
Non-specific test	Letieri et al.[45]B	Combined	-1.485	0.478	-2.422	-0.548
Non-specific test	Martin-Hernandez et al.[12]A-ns	Combined	-0.722	0.435	-1.574	0.130
Non-specific test	Martin-Hernandez et al.[12]B-ns	Combined	-0.306	0.422	-1.134	0.521
Non-specific test	Mendonca et al.[50]	mvc	-0.023	0.355	-0.719	0.674
Non-specific test	Morley et al.[51]A-ns	mvc	0.291	0.503	-0.695	1.278
Non-specific test	Morley et al.[51]B-ns	mvc	0.217	0.519	-0.801	1.235
Non-specific test	Ramis et al.[52]	Combined	-1.071	0.400	-1.856	-0.287
Non-specific test	Sousa et al.[55]	mvc	-1.355	0.469	-2.274	-0.437
Non-specific test	Sugiarto et al.[56]	Combined	0.877	0.563	-0.228	1.981
Non-specific test	Yasuda et al.[11]-ns	mvc	-0.979	0.455	-1.872	-0.086
Non-specific test			-0.422	0.120	-0.657	-0.188
Specific test	Buckner et al.[32]A-s	1rm	-1.141	0.335	-1.798	-0.484
Specific test	Buckner et al.[32]B-s	1rm	-0.955	0.328	-1.598	-0.313
Specific test	Centner et al.[28]	Combined	0.945	0.421	0.119	1.771
Specific test	Centner et al.[65]	1rm	0.208	0.362	-0.502	0.918
Specific test	Cook et al.[34]-s	Combined	-0.706	0.410	-1.509	0.097
Specific test	Cook et al.[35]-s	1rm	-0.734	0.554	-1.819	0.351
Specific test	De Araujo et al.[66]	1rm	-1.895	0.523	-2.920	-0.871
Specific test	de Lemos Muller et al.[37]	Combined	-1.894	0.479	-2.834	-0.955
Specific test	Ellefsen et al.[17]	1rm	-0.327	0.397	-1.106	0.451
Specific test	Horiuchi et al.[67]	Combined	-0.465	0.401	-1.250	0.320
Specific test	Jessee et al.[39]A-s	1rm	-1.615	0.499	-2.593	-0.638
Specific test	Jessee et al.[39]B-s	1rm	-1.303	0.475	-2.234	-0.371
Specific test	Karabulut et al.[9]	Combined	-0.263	0.396	-1.040	0.514
Specific test	Kim et al.[40]	Combined	-0.618	0.442	-1.485	0.248
Specific test	Kim et al.[41]	1rm	-0.324	0.452	-1.211	0.562
Specific test	Laurentino et al.[15]	1rm	0.840	0.460	-0.061	1.741
Specific test	Libardi et al.[46]	1rm	-0.832	0.473	-1.758	0.094
Specific test	Lixandrao et al.[47]A	1rm	-1.087	0.464	-1.995	-0.178
Specific test	Lixandrao et al.[47]B	1rm	-0.982	0.437	-1.837	-0.126
Specific test	Lixandrao et al.[47]C	1rm	-0.871	0.485	-1.821	0.079
Specific test	Lixandrao et al.[47]D	1rm	-0.852	0.460	-1.753	0.050
Specific test	Martin-Hernandez et al.[12]A-s	1rm	-1.489	0.478	-2.426	-0.552
Specific test	Martin-Hernandez et al.[12]B-s	1rm	-1.502	0.479	-2.441	-0.563
Specific test	May et al.[49]	Combined	-0.186	0.463	-1.093	0.721
Specific test	Morley et al.[51]A-s	1rm	-1.045	0.538	-2.100	0.009
Specific test	Morley et al.[51]B-s	1rm	-0.411	0.524	-1.438	0.615
Specific test	Ozaki et al.[16]	1rm	-0.868	0.461	-1.772	0.035
Specific test	Reece et al.[69]A	1rm	-1.883	0.614	-3.087	-0.680
Specific test	Reece et al.[69]B	f1rm	-0.899	0.499	-1.876	0.079
Specific test	Sharifi et al.[53]A	Combined	-0.285	0.475	-1.216	0.647
Specific test	Sharifi et al.[53]B	Combined	-0.086	0.541	-1.147	0.974
Specific test	Shiromaru et al.[54]	1rm	-0.524	0.362	-1.233	0.185
Specific test	Sousa-Silva et al.[70]	1rm	-0.734	0.465	-1.646	0.178
Specific test	Teixeira et al.[57]	1rm	-0.501	0.481	-1.444	0.441
Specific test	Thiebaud et al.[58]	Combined	-0.469	0.523	-1.494	0.556
Specific test	Vechin et al.[13]	1rm	-0.692	0.488	-1.649	0.265
Specific test	Yasuda et al.[11]-s	1rm	-1.089	0.462	-1.993	-0.184
Specific test			-0.715	0.099	-0.909	-0.522

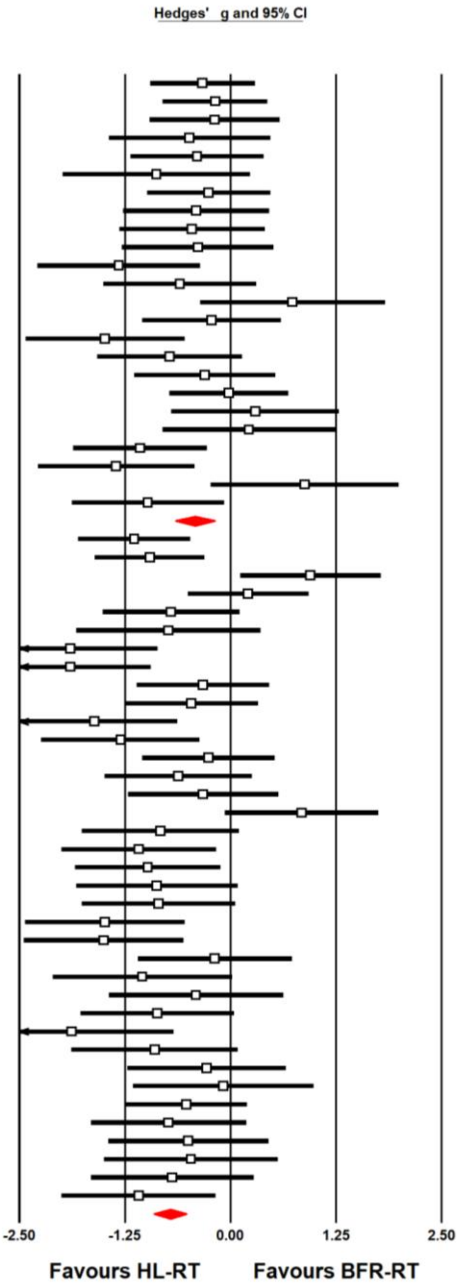

Fig. S12 Funnel plot of studies comparing muscle strength between high-load resistance training (HL-RT) vs. low-load resistance training combined with blood-flow restriction (BFR-RT). The diamond represents the Hedges' g overall standard error.

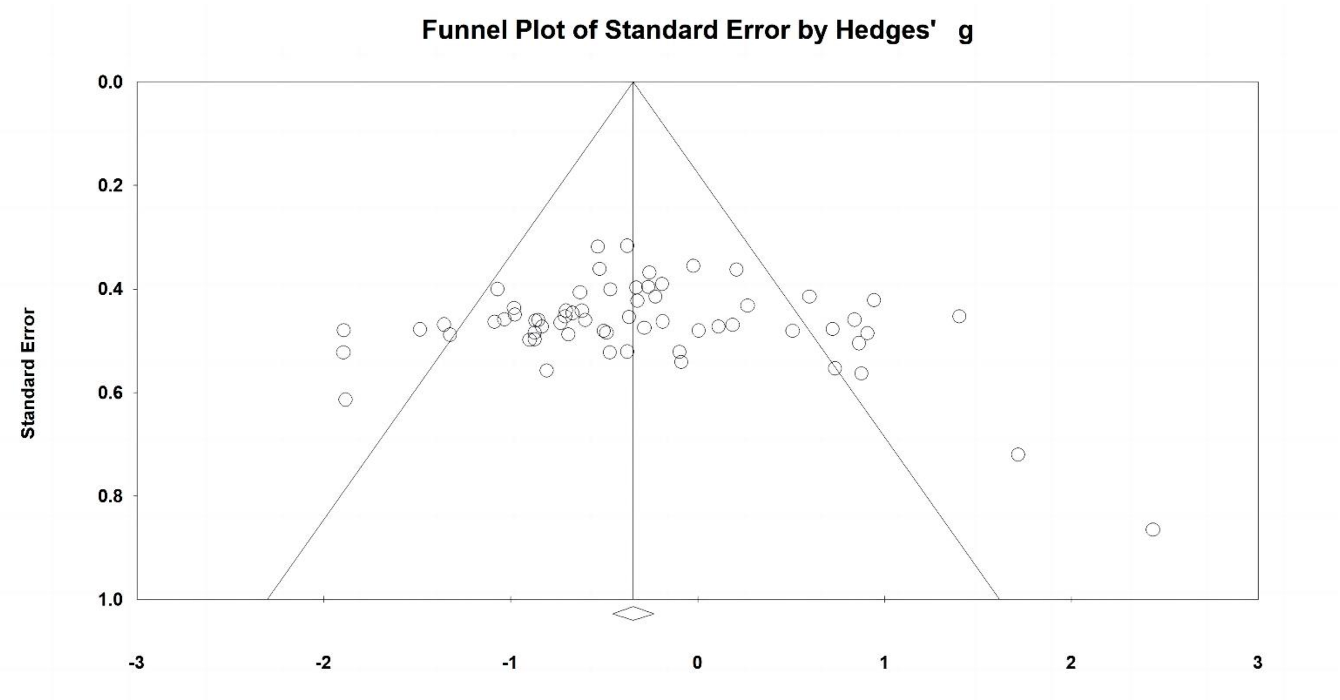

Fig. S13 Forest plot of the effect size difference between BFR-RT versus HL-RT for muscle hypertrophy according to age in the untrained individuals. The different capital letters (i.e. A, B, C, D) after the reference number are used to represent different training protocols for the same study. Hedges'g represents effect size difference. Red diamonds represent overall Hedges'g of subgroups. *BFR-RT* blood-flow restriction low-load resistance training, *CI* confidence interval, *Combined* mean of multiple outcomes from the same training protocol, *csa* cross-section area, *HL-RT* high-load resistance training, *mt* muscle thickness.

Group by AGE	Study name	Outcome	Statistics for each study: Hedges' g	Standard error	Lower limit	Upper limit
Old	Cook et al.[34]	csa	0.229	0.396	-0.546	1.005
Old	Libardi et al.[46]	csa	-0.026	0.452	-0.911	0.860
Old	Thiebaud et al.[58]	Combined	0.164	0.507	-0.829	1.158
Old	Vechin et al.[13]	csa	-0.020	0.473	-0.947	0.906
Old			0.096	0.226	-0.346	0.538
Young	Buckner et al.[32]A	Combined	-0.666	0.319	-1.292	-0.040
Young	Buckner et al.[32]B	Combined	-0.414	0.316	-1.034	0.206
Young	Centner et al.[28]	Combined	-0.367	0.367	-1.085	0.352
Young	Centner et al.[33]	csa	0.238	0.391	-0.529	1.004
Young	Cook et al.[35]	csa	-0.143	0.534	-1.189	0.903
Young	Ellefsen et al.[17]	Combined	-0.276	0.397	-1.053	0.501
Young	Jessee et al.[39]A	Combined	-0.286	0.433	-1.135	0.563
Young	Jessee et al.[39]B	Combined	-0.226	0.432	-1.073	0.622
Young	Kataoka et al.[63]	Combined	0.492	0.273	-0.043	1.028
Young	Kim et al.[41]	Combined	0.032	0.449	-0.849	0.913
Young	Kim et al.[64]	csa	-0.113	0.429	-0.954	0.727
Young	Kubo et al.[10]	csa	0.063	0.449	-0.817	0.944
Young	Laurentino et al.[15]	csa	-0.263	0.441	-1.128	0.601
Young	Lixandrao et al.[47]A	csa	-0.661	0.443	-1.530	0.207
Young	Lixandrao et al.[47]B	csa	-0.449	0.417	-1.266	0.369
Young	Lixandrao et al.[47]C	csa	-0.233	0.463	-1.141	0.674
Young	Lixandrao et al.[47]D	csa	-0.069	0.439	-0.929	0.792
Young	Martin-Hernandez et al.[12]A	Combined	0.180	0.420	-0.644	1.004
Young	Martin-Hernandez et al.[12]B	Combined	0.032	0.420	-0.790	0.854
Young	May et al.[49]	Combined	0.142	0.463	-0.765	1.048
Young	Mendonca et al.[50]	csa	-0.289	0.357	-0.989	0.411
Young	Ozaki et al.[16]	csa	-0.474	0.446	-1.347	0.399
Young	Ramis et al.[52]	Combined	-0.244	0.370	-0.969	0.482
Young	Shiromaru et al.[54]	Combined	0.070	0.368	-0.652	0.791
Young	Sousa-Silva et al.[70]	mt	-0.102	0.449	-0.983	0.778
Young	Teixeira et al.[57]	csa	0.053	0.473	-0.874	0.980
Young	Yasuda et al.[11]	csa	-0.452	0.434	-1.303	0.399
Young			-0.155	0.077	-0.306	-0.003

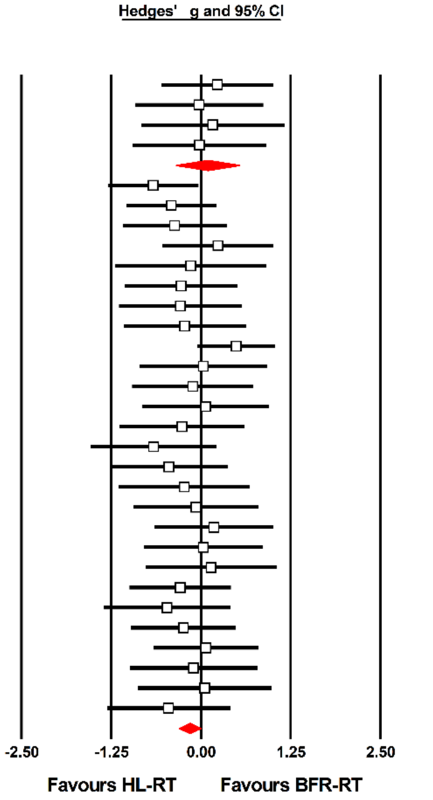

Fig. S14 Forest plot of the effect size difference between BFR-RT versus HL-RT for muscle hypertrophy according to limbs in the untrained individuals. The different capital letters (i.e. A, B, C, D) after the reference number are used to represent different training protocols for the same study. Hedges'g represents effect size difference. Red diamonds represent overall Hedges'g of subgroups. *BFR-RT* blood-flow restriction low-load resistance training, *CI* confidence interval, *Combined* mean of multiple outcomes from the same training protocol, *csa* cross-section area, *HL-RT* high-load resistance training, *mas* muscle mass, *mt* muscle thickness.

Group by LIMBS	Study name	Outcome	Statistics for each study			
			Hedges' g	Standard error	Lower limit	Upper limit
Lower limbs	Centner et al.[28]	Combined	-0.367	0.367	-1.085	0.352
Lower limbs	Centner et al.[33]	csa	0.238	0.391	-0.529	1.004
Lower limbs	Cook et al.[34]	csa	0.229	0.396	-0.546	1.005
Lower limbs	Cook et al.[35]	mas	-0.143	0.534	-1.189	0.903
Lower limbs	Ellefsen et al.[17]	Combined	-0.276	0.397	-1.053	0.501
Lower limbs	Jessee et al.[39]A	Combined	-0.286	0.433	-1.135	0.563
Lower limbs	Jessee et al.[39]B	Combined	-0.226	0.432	-1.073	0.622
Lower limbs	Kataoka et al.[63]	Combined	0.492	0.273	-0.043	1.028
Lower limbs	Kim et al.[64]	csa	-0.113	0.429	-0.954	0.727
Lower limbs	Kubo et al.[10]	mas	0.063	0.449	-0.817	0.944
Lower limbs	Laurentino et al.[15]	csa	-0.263	0.441	-1.128	0.601
Lower limbs	Libardi et al.[46]	csa	-0.026	0.452	-0.911	0.860
Lower limbs	Lixandrao et al.[47]A	csa	-0.661	0.443	-1.530	0.207
Lower limbs	Lixandrao et al.[47]B	csa	-0.449	0.417	-1.266	0.369
Lower limbs	Lixandrao et al.[47]C	csa	-0.233	0.463	-1.141	0.674
Lower limbs	Lixandrao et al.[47]D	csa	-0.069	0.439	-0.929	0.792
Lower limbs	Martin-Hernandez et al.[12]A	Combined	0.180	0.420	-0.644	1.004
Lower limbs	Martin-Hernandez et al.[12]B	Combined	0.032	0.420	-0.790	0.854
Lower limbs	May et al.[49]	Combined	0.142	0.463	-0.765	1.048
Lower limbs	Mendonca et al.[50]	csa	-0.289	0.357	-0.989	0.411
Lower limbs	Ramis et al.[52]	mt	-0.081	0.368	-0.802	0.640
Lower limbs	Shiromaru et al.[54]	Combined	0.070	0.368	-0.652	0.791
Lower limbs	Teixeira et al.[57]	csa	0.053	0.473	-0.874	0.980
Lower limbs	Vechin et al.[13]	csa	-0.020	0.473	-0.947	0.906
Lower limbs			-0.053	0.084	-0.217	0.111
Upper limbs	Buckner et al.[32]A	Combined	-0.666	0.319	-1.292	-0.040
Upper limbs	Buckner et al.[32]B	Combined	-0.414	0.316	-1.034	0.206
Upper limbs	Kim et al.[41]	Combined	0.032	0.449	-0.849	0.913
Upper limbs	Ozaki et al.[16]	csa	-0.474	0.446	-1.347	0.399
Upper limbs	Ramis et al.[52]	mt	-0.406	0.372	-1.135	0.323
Upper limbs	Sousa-Silva et al.[70]	mt	-0.102	0.449	-0.983	0.778
Upper limbs	Thiebaud et al.[58]	Combined	0.164	0.507	-0.829	1.158
Upper limbs	Yasuda et al.[11]	csa	-0.452	0.434	-1.303	0.399
Upper limbs			-0.354	0.140	-0.628	-0.080

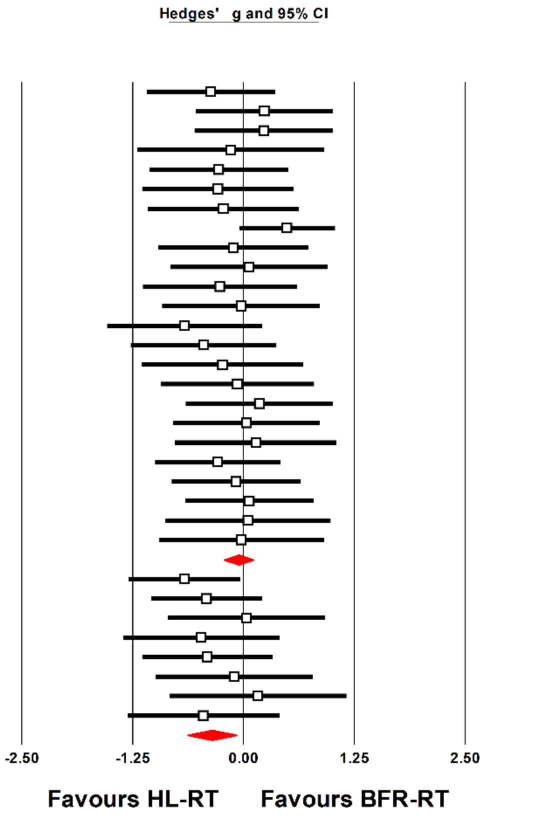

Fig. S15 Forest plot of the effect size difference between BFR-RT versus HL-RT for muscle hypertrophy according to training duration in the untrained individuals. The different capital letters (i.e. A, B, C, D) after the reference number are used to represent different training protocols for the same study. Hedges'g represents effect size difference. Red diamonds represent overall Hedges'g of subgroups. *BFR-RT* blood-flow restriction low-load resistance training, *CI* confidence interval, *Combined* mean of multiple outcomes from the same training protocol, *csa* cross-section area, *HL-RT* high-load resistance training, *mt* muscle thickness.

Group by DURATION	Study name	Outcome	Statistics for each study: Hedges' g	Standard error	Lower limit	Upper limit
<=4wk	Kim et al.[64]	csa	-0.113	0.429	-0.954	0.727
<=4wk	Mendonca et al.[50]	csa	-0.289	0.357	-0.989	0.411
<=4wk	Shiromaru et al.[54]	Combined	0.070	0.368	-0.652	0.791
<=4wk			-0.115	0.220	-0.546	0.317
=5-8wk	Buckner et al.[32]A	Combined	-0.666	0.319	-1.292	-0.040
=5-8wk	Buckner et al.[32]B	Combined	-0.414	0.316	-1.034	0.206
=5-8wk	Cook et al.[34]	csa	0.229	0.396	-0.546	1.005
=5-8wk	Cook et al.[35]	csa	-0.143	0.534	-1.189	0.903
=5-8wk	Jessee et al.[39]A	Combined	-0.286	0.433	-1.135	0.563
=5-8wk	Jessee et al.[39]B	Combined	-0.226	0.432	-1.073	0.622
=5-8wk	Kataoka et al.[63]	Combined	0.492	0.273	-0.043	1.028
=5-8wk	Kim et al.[41]	Combined	0.032	0.449	-0.849	0.913
=5-8wk	Laurentino et al.[15]	csa	-0.263	0.441	-1.128	0.601
=5-8wk	Martin-Hernandez et al.[12]A	Combined	0.180	0.420	-0.644	1.004
=5-8wk	Martin-Hernandez et al.[12]B	Combined	0.032	0.420	-0.790	0.854
=5-8wk	May et al.[49]	Combined	0.142	0.463	-0.765	1.048
=5-8wk	Ozaki et al.[16]	csa	-0.474	0.446	-1.347	0.399
=5-8wk	Ramis et al.[52]	Combined	-0.244	0.370	-0.969	0.482
=5-8wk	Sousa-Silva et al.[70]	mt	-0.102	0.449	-0.983	0.778
=5-8wk	Teixeira et al.[57]	csa	0.053	0.473	-0.874	0.980
=5-8wk	Thiebaud et al.[58]	Combined	0.164	0.507	-0.829	1.158
=5-8wk	Yasuda et al.[11]	csa	-0.452	0.434	-1.303	0.399
=5-8wk			-0.102	0.095	-0.289	0.084
>=9 wk	Centner et al.[28]	Combined	-0.367	0.367	-1.085	0.352
>=9 wk	Centner et al.[33]	csa	0.238	0.391	-0.529	1.004
>=9 wk	Ellefsen et al.[17]	Combined	-0.276	0.397	-1.053	0.501
>=9 wk	Kubo et al.[10]	csa	0.063	0.449	-0.817	0.944
>=9 wk	Libardi et al.[46]	csa	-0.026	0.452	-0.911	0.860
>=9 wk	Lixandrao et al.[47]A	csa	-0.661	0.443	-1.530	0.207
>=9 wk	Lixandrao et al.[47]B	csa	-0.449	0.417	-1.266	0.369
>=9 wk	Lixandrao et al.[47]C	csa	-0.233	0.463	-1.141	0.674
>=9 wk	Lixandrao et al.[47]D	csa	-0.069	0.439	-0.929	0.792
>=9 wk	Vechin et al.[13]	csa	-0.020	0.473	-0.947	0.906
>=9 wk			-0.185	0.134	-0.448	0.079

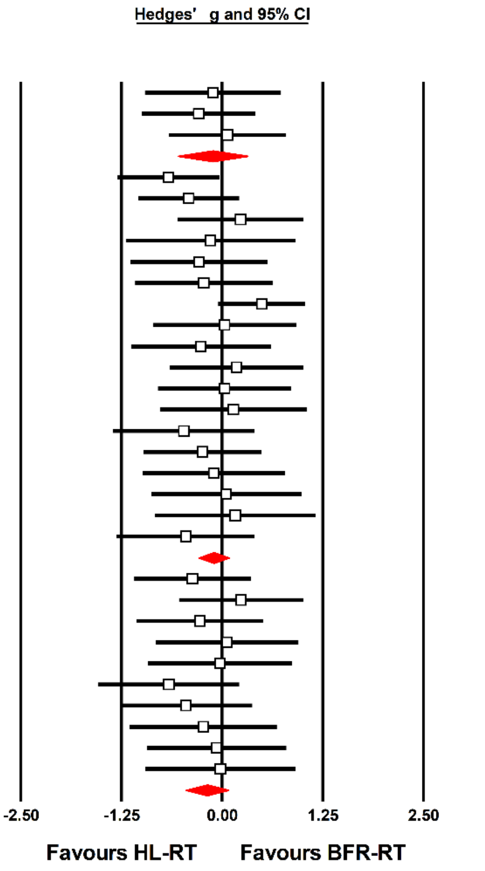

Fig. S16 Forest plot of the effect size difference between BFR-RT versus HL-RT for muscle hypertrophy according to training frequency in the untrained individuals. The different capital letters (i.e. A, B, C, D) after the reference number are used to represent different training protocols for the same study. Hedges'g represents effect size difference. Red diamonds represent overall Hedges'g of subgroups. *BFR-RT* blood-flow restriction low-load resistance training, *CI* confidence interval, *Combined* mean of multiple outcomes from the same training protocol, *csa* cross-section area, *HL-RT* high-load resistance training, *mt* muscle thickness.

Group by FREQUENCY	Study name	Outcome	Statistics for each study			
			Hedges' g	Standard error	Lower limit	Upper limit
2/wk	Buckner et al.[32]A	Combined	-0.666	0.319	-1.292	-0.040
2/wk	Buckner et al.[32]B	Combined	-0.414	0.316	-1.034	0.206
2/wk	Cook et al.[34]	csa	0.229	0.396	-0.546	1.005
2/wk	Ellefsen et al.[17]	Combined	-0.276	0.397	-1.053	0.501
2/wk	Jessee et al.[39]A	Combined	-0.286	0.433	-1.135	0.563
2/wk	Jessee et al.[39]B	Combined	-0.226	0.432	-1.073	0.622
2/wk	Laurentino et al.[15]	csa	-0.263	0.441	-1.128	0.601
2/wk	Libardi et al.[46]	csa	-0.026	0.452	-0.911	0.860
2/wk	Lixandrao et al.[47]A	csa	-0.661	0.443	-1.530	0.207
2/wk	Lixandrao et al.[47]B	csa	-0.449	0.417	-1.266	0.369
2/wk	Lixandrao et al.[47]C	csa	-0.233	0.463	-1.141	0.674
2/wk	Lixandrao et al.[47]D	csa	-0.069	0.439	-0.929	0.792
2/wk	Martin-Hernandez et al.[12]A	Combined	0.180	0.420	-0.644	1.004
2/wk	Martin-Hernandez et al.[12]B	Combined	0.032	0.420	-0.790	0.854
2/wk	Sousa-Silva et al.[70]	mt	-0.102	0.449	-0.983	0.778
2/wk	Teixeira et al.[57]	csa	0.053	0.473	-0.874	0.980
2/wk	Vechin et al.[13]	csa	-0.020	0.473	-0.947	0.906
2/wk			-0.219	0.100	-0.416	-0.022
3/wk	Centner et al.[28]	Combined	-0.367	0.367	-1.085	0.352
3/wk	Centner et al.[33]	csa	0.238	0.391	-0.529	1.004
3/wk	Cook et al.[35]	csa	-0.143	0.534	-1.189	0.903
3/wk	Kataoka et al.[63]	Combined	0.492	0.273	-0.043	1.028
3/wk	Kim et al.[41]	Combined	0.032	0.449	-0.849	0.913
3/wk	Kim et al.[64]	csa	-0.113	0.429	-0.954	0.727
3/wk	Kubo et al.[10]	csa	0.063	0.449	-0.817	0.944
3/wk	May et al.[49]	Combined	0.142	0.463	-0.765	1.048
3/wk	Ozaki et al.[16]	csa	-0.474	0.446	-1.347	0.399
3/wk	Ramis et al.[52]	Combined	-0.244	0.370	-0.969	0.482
3/wk	Shiromaru et al.[54]	Combined	0.070	0.368	-0.652	0.791
3/wk	Thiebaud et al.[58]	Combined	0.164	0.507	-0.829	1.158
3/wk	Yasuda et al.[11]	csa	-0.452	0.434	-1.303	0.399
3/wk			0.000	0.112	-0.219	0.220

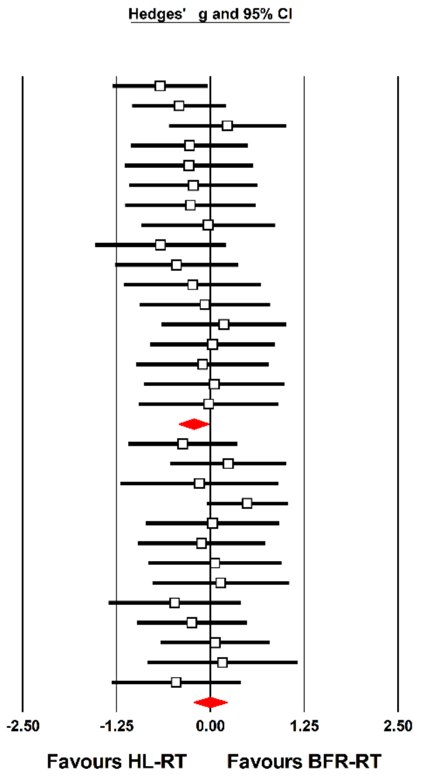

Fig. S17 Forest plot of the effect size difference between BFR-RT versus HL-RT for muscle hypertrophy according to lower limb region specificity in the untrained individuals. The different capital letters (i.e. A, B, C, D) after the reference number are used to represent different training protocols for the same study. Hedges'g represents effect size difference. Red diamonds represent overall Hedges'g of subgroups. *BFR-RT* blood-flow restriction low-load resistance training, *CI* confidence interval, *Combined* mean of multiple outcomes from the same training protocol, *csa* cross-section area, *HL-RT* high-load resistance training, *-d* distal femur, *-m* middle femur, *-p* proximal femur.

Group by REGION SPECIFICITY	Study name	Outcome	Statistics for each study			
			Hedges' g	Standard error	Lower limit	Upper limit
Distal	Centner et al.[28]-d	Combined	-0.066	0.362	-0.776	0.645
Distal	Ellefsen et al.[17]-d	csa	-0.145	0.395	-0.919	0.629
Distal	Jessee et al.[39]A-d	Combined	0.000	0.429	-0.841	0.841
Distal	Jessee et al.[39]B-d	Combined	-0.249	0.430	-1.093	0.594
Distal	May et al.[49]-d	Combined	0.157	0.463	-0.750	1.065
Distal			-0.069	0.184	-0.430	0.291
Mid	Centner et al.[28]-m	csa	-0.445	0.366	-1.162	0.272
Mid	Jessee et al.[39]A-m	Combined	-0.136	0.429	-0.977	0.704
Mid	Jessee et al.[39]B-m	Combined	-0.309	0.432	-1.155	0.537
Mid	Kim et al.[64]	csa	-0.113	0.429	-0.954	0.727
Mid	Laurentino et al.[15]	csa	-0.263	0.441	-1.128	0.601
Mid	Libardi et al.[46]	csa	-0.026	0.452	-0.911	0.860
Mid	Lixandrao et al.[47]A-m	csa	-0.661	0.443	-1.530	0.207
Mid	Lixandrao et al.[47]B-m	csa	-0.449	0.417	-1.266	0.369
Mid	Lixandrao et al.[47]C-m	csa	-0.233	0.463	-1.141	0.674
Mid	Lixandrao et al.[47]D-m	csa	-0.069	0.439	-0.929	0.792
Mid	Martin-Hernandez et al.[12]A	Combined	0.032	0.420	-0.790	0.854
Mid	Martin-Hernandez et al.[12]B	Combined	0.180	0.420	-0.644	1.004
Mid	May et al.[49]-m	csa	0.095	0.461	-0.810	0.999
Mid	Shiromaru et al.[54]	Combined	0.070	0.368	-0.652	0.791
Mid	Teixeira et al.[57]	csa	0.053	0.473	-0.874	0.980
Mid	Vechin et al.[13]	csa	-0.020	0.473	-0.947	0.906
Mid			-0.149	0.107	-0.359	0.062
Proximal	Centner et al.[28]-p	Combined	-0.541	0.370	-1.266	0.183
Proximal	Ellefsen et al.[17]-p	csa	-0.407	0.399	-1.188	0.374
Proximal	Jessee et al.[39]A-p	Combined	-0.504	0.437	-1.360	0.353
Proximal	Jessee et al.[39]B-p	Combined	-0.173	0.434	-1.023	0.678
Proximal			-0.417	0.203	-0.816	-0.018

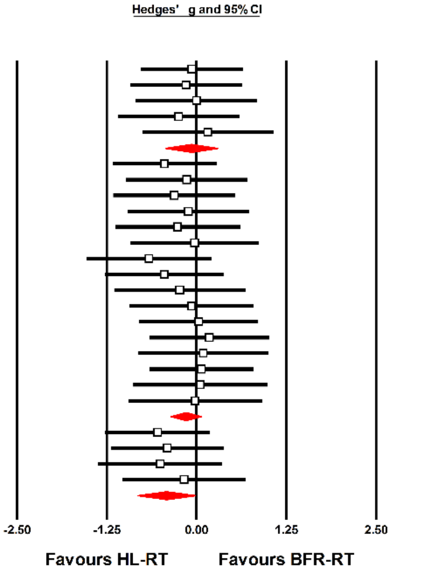

Fig. S18 Forest plot of the effect size difference between BFR-RT versus HL-RT for muscle hypertrophy according to upper limb region specificity in the untrained individuals. The different capital letters (i.e. A, B) after the reference number are used to represent different training protocols for the same study. Hedges'g represents effect size difference. Red diamonds represent overall Hedges'g of subgroups. *BFR-RT* blood-flow restriction low-load resistance training, *CI* confidence interval, *Combined* mean of multiple outcomes from the same training protocol, *csa* cross-section area, *HL-RT* high-load resistance training, *mt* muscle thickness, *-m* middle humerus, *-p* proximal humerus.

Group by REGION SPECIFICITY	Study name	Outcome	Statistics for each study			
			Hedges' g	Standard error	Lower limit	Upper limit
Distal	Buckner et al.[32]A-d	Combined	-0.549	0.316	-1.168	0.071
Distal	Buckner et al.[32]B-d	Combined	-0.269	0.314	-0.885	0.348
Distal	Kim et al.[41]-d	Combined	-0.030	0.449	-0.910	0.850
Distal	Sousa-Silva et al.[70]	mt	-0.102	0.449	-0.983	0.778
Distal	Thiebaud et al.[58]	Combined	0.164	0.507	-0.829	1.158
Distal			-0.242	0.172	-0.579	0.094
Mid	Buckner et al.[32]A-m	mt	-0.900	0.326	-1.539	-0.262
Mid	Buckner et al.[32]B-m	mt	-0.703	0.320	-1.330	-0.077
Mid	Kim et al.[41]-m	mt	0.156	0.450	-0.725	1.037
Mid	Yasuda et al.[11]	csa	-0.452	0.434	-1.303	0.399
Mid			-0.577	0.184	-0.938	-0.216

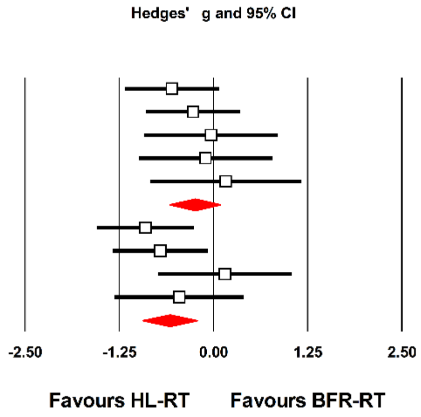

Fig. S19 Funnel plot of studies comparing muscle hypertrophy between high-load resistance training (HL-RT) vs. low-load resistance training combined with blood-flow restriction (BFR-RT). The diamond represents the Hedges' g overall standard error.

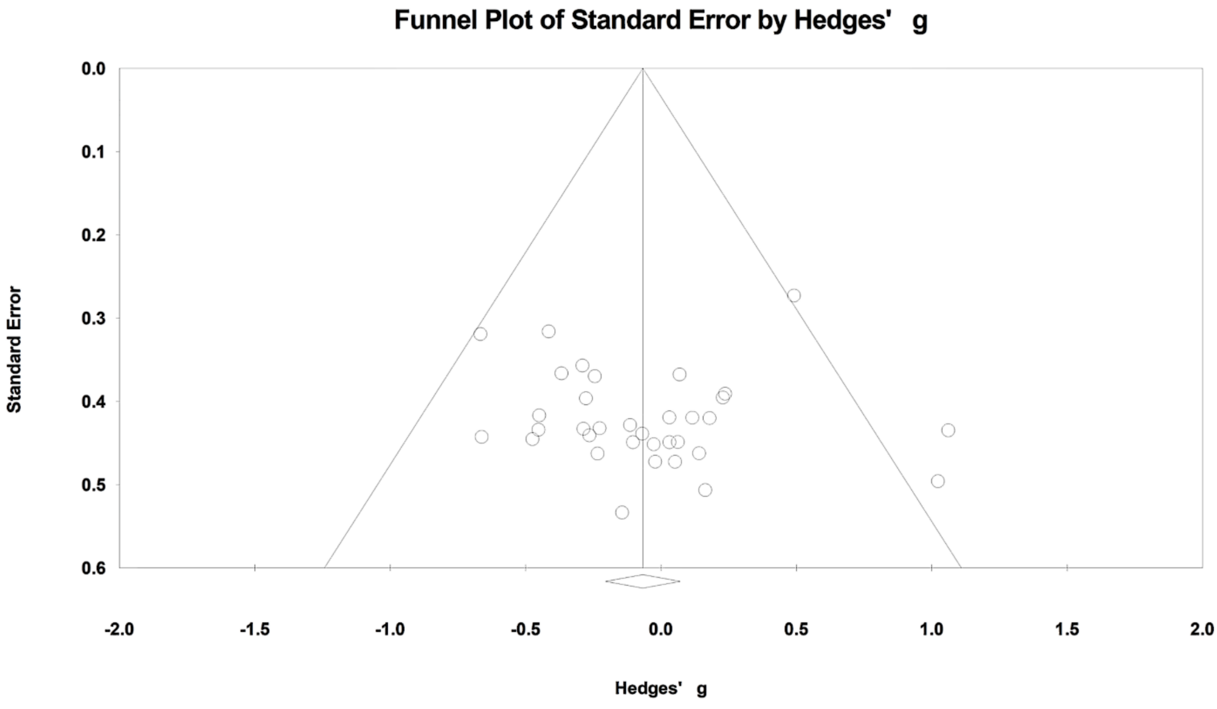

Supplement: Supplementary file 1 — Additional file 1 [file 40798_2024_719_MOESM1_ESM.pdf]
